# Effective prediction of short hydrogen bonds in proteins via machine learning method

**DOI:** 10.1038/s41598-021-04306-4

**Published:** 2022-01-10

**Authors:** Shengmin Zhou, Yuanhao Liu, Sijian Wang, Lu Wang

**Affiliations:** 1 Department of Chemistry and Chemical Biology, Institute for Quantitative Biomedicine, Rutgers University, Piscataway, NJ 08854 USA; 2 Department of Statistics, Institute for Quantitative Biomedicine, Rutgers University, Piscataway, NJ 08854 USA

**Keywords:** Computational chemistry, Quantum chemistry, Structure prediction, Biophysical chemistry

## Abstract

Short hydrogen bonds (SHBs), whose donor and acceptor heteroatoms lie within 2.7 Å, exhibit prominent quantum mechanical characters and are connected to a wide range of essential biomolecular processes. However, exact determination of the geometry and functional roles of SHBs requires a protein to be at atomic resolution. In this work, we analyze 1260 high-resolution peptide and protein structures from the Protein Data Bank and develop a boosting based machine learning model to predict the formation of SHBs between amino acids. This model, which we name as machine learning assisted prediction of short hydrogen bonds (MAPSHB), takes into account 21 structural, chemical and sequence features and their interaction effects and effectively categorizes each hydrogen bond in a protein to a short or normal hydrogen bond. The MAPSHB model reveals that the type of the donor amino acid plays a major role in determining the class of a hydrogen bond and that the side chain Tyr-Asp pair demonstrates a significant probability of forming a SHB. Combining electronic structure calculations and energy decomposition analysis, we elucidate how the interplay of competing intermolecular interactions stabilizes the Tyr-Asp SHBs more than other commonly observed combinations of amino acid side chains. The MAPSHB model, which is freely available on our web server, allows one to accurately and efficiently predict the presence of SHBs given a protein structure with moderate or low resolution and will facilitate the experimental and computational refinement of protein structures.

## Introduction

The three-dimensional architecture of proteins often creates specialized structural elements, notably hydrogen bonds that are much shorter than those commonly observed in liquids. These short hydrogen bonds (SHBs) have the donor-acceptor separations, R, below 2.7 Å, and are frequently observed to connect amino acids in the backbone and side chains of proteins and link amino acids and ligands in the active site of enzymes^1–5^. Compared to a normal hydrogen bond (NHB), whose R lies between 2.8 and 3.2 Å^6^, the donor and acceptor atoms in a SHB reside considerably closer than the sum of their van der Waals radii. As such, SHBs exhibit prominent covalent characters and electronic quantum effects such as charge transfer and dispersion play an important role in stabilizing these compact structures^7–9^. As SHBs are a broad concept that encompasses low-barrier hydrogen bonds (LBHBs), whose proton potential energy surfaces take the shape of a double well with a low barrier, and single-well hydrogen bonds with vanishing proton transfer barrier^4,10,11^, nuclear quantum effects such as zero-point energies and tunneling can also dramatically alter their behavior^12–15^. The quantum nature of SHBs can manifest as distinctive geometric and spectroscopic properties, including shared protons between the heteroatoms, significantly red shifted Donor–H stretch frequencies, negative anharmonicity in the vibrational spectra, far downfield $^1$H NMR chemical shifts and large isotope effects when hydrogen is substituted by deuterium^7,8,10,11,16–22^.

An intriguing class of SHBs is LBHBs, also known as short strong hydrogen bonds, for which R is around 2.5 Å and the $pK_a$ of the heteroatoms are closely matched^23^. At these short distances, the zero-point energy of an O–H or N–H vibration, which is approximately 5 kcal/mol, becomes comparable to the proton transfer barrier and promotes the quantum delocalization of the proton between the donor and acceptor atoms. LBHBs have been associated with a variety of biology functions, ranging from maintaining the native structures of proteins to stabilizing the transition states in enzymatic reactions and facilitating signaling in biological systems^3,11,18,24–33^. Although their geometries and functional importance are still under debate^34–39^, advancements in experimental and computational techniques have provided new insights into this problem. For example, Tittmann and coworkers have recently determined a sub-angstrom resolution X-ray structure of human transketolase with the bound thiamine diphosphate cofactors, and identified a LBHB between the side chains of Glu160 and the catalytic residue Glu366′ that reside in two neighboring subunits of the protein^32^. The LBHB is part of an extended hydrogen bond network that links a few key amino acids with the cofactors and water molecules, forming a communication channel between the distant active sites of the two subunits. The electron density maps show that the LBHB has an R of 2.56 Å and the proton is located almost exactly halfway between the O atoms. Site-directed mutagenesis studies suggest that this LBHB allows for an efficient proton transfer along the hydrogen bond network and a cooperative signal transduction between the distant active sites of the protein^32^.

Despite their importance, pinpointing biological SHBs requires a protein structure at atomic resolution (< 1.2 Å), which poses a challenge to structure determination techniques such as X-ray crystallography, NMR spectroscopy and electron microscopy. For example, the coordinate error in a well-ordered protein structure with a 2 Å resolution is about 0.2 Å, which gives a significant uncertainty of 0.3 Å in R^33^. While force field based minimization and computational structure predictions have greatly improved the crystallographic refinement process^40–42^, the relevant classical force fields cannot fully account for the quantum nature of the SHBs and their van der Waals parameters would push the length of a hydrogen bond to $\sim$3 Å. Due to the coordinate uncertainty in the crystal structures and the inaccuracy of classical force fields, it is often difficult to determine the presence of SHBs in a biological macromolecule. As such, it is highly desirable to develop a method that allows for an effective prediction of SHBs based on a protein structure with moderate or low resolution.

In a recent study, Zhou and Wang have analyzed the top 1% highest-quality macromolecular structures in the Protein Data Bank (PDB)^43^ and characterized the preferred location, geometry and amino acid composition of SHBs^4^. We find that the side chains of amino acids are particularly important as they act as donors in over 93% of all biological SHBs. The most common donors are the neutral residues Ser, Thr and Tyr, while the predominant acceptors are the anionic Asp and Glu. When a backbone residue becomes an acceptor, it can reside in ordered secondary structures such as $\alpha$-helices and $\beta$-sheets as well as in disordered regions such as coils and turns^4^. The interplay of the geometric and chemical features helps position the hydrogen bonded atoms in close proximity in the interior or on the surface of proteins, making it more likely to observe SHBs in these biological molecules than in small molecules and simple liquids. Combining these features and the sequence information, in this work, we consider 1260 high-resolution peptide and protein structures from the PDB and develop a machine learning model that effectively predicts the formation of SHBs between amino acids. This model is based on the boosting method and utilizes the undersampling strategy. We then reveal the key factors that facilitate the formation of SHBs and unravel why the phenol side chain of Tyr and the carboxylate side chains of Asp and Glu have a significant probability of forming such close contacts.

## Results and discussion

### Dataset for biological hydrogen bonds

From the PDB, we obtain 2171 peptide and protein structures that are determined from X-ray or neutron scattering experiments and have a resolution equal to or higher than 1.1 Å. 35% of these structures are small proteins with fewer than 150 amino acids, and 12% of them are peptides containing less than 50 amino acids. To validate these structures, we find that 98.2% of them have an *R*-factor $\le$ 0.20 and the difference between the values of *R*-free and *R*-factor $\le$ 7%, confirming that these are high-quality crystal structures^44^. The remaining protein structures have a slightly larger *R*-factor between 0.21 and 0.28. We note that some proteins have multiple PDB entries for their wild-type, mutated and ligand-bound structures. To construct the training set of the machine learning model, we remove the “redundant” entries and include 782 structures that have unique protein names. The test set contains 478 peptides and proteins with the structural redundancy retained.

From the 1260 protein structures in the training and test datasets, we collect a total of 10161 SHBs and 44871 NHBs that form between amino acids and have the side chain of an amino acid as the donor group. As shown in Table S1, the probability of finding a SHB is 24% and 13% for a side chain-side chain and side chain-backbone hydrogen bond, respectively. In contrast, this probability reduces to 0.4% when the donor group is in the protein backbone, and hence we preclude these cases. A pair A–H$\cdot \cdot \cdot$B is treated as a SHB or NHB if it satisfies all of the following criteria: (1) the heteroatoms are N or O; (2) 2.3 Å $\le R\le$ 2.7 Å for a SHB, and 2.8 Å $\le R\le$ 3.2 Å for a NHB; (3) the A–H–B angle $\ge$ 135$^\circ$. Here we don’t consider other types of hydrogen bonds such as those involving S or C atoms, and we use a separation of 0.1 Å in R to better distinguish a SHB and a NHB. This analysis yields 6181 SHBs and 26929 NHBs in the training set, and 3980 SHBs and 17942 NHBs in the test set. Therefore, the probability of observing SHBs in the training and test sets are 18.7% and 18.2%, respectively.

### Machine learning model for the prediction of SHBs in proteins

Based on a recent statistical analysis of the PDB^4^, we use 21 variables that cover the structural, chemical and sequence information of SHBs as the input features of the machine learning model. These include the residues and heteroatoms involved in the formation of hydrogen bonds, the charge, location (backbone or side chain) and secondary structure of the donor and acceptor groups, and the sequence of amino acids that are within 3 residues before and after the hydrogen bonded residues. We only consider N and O as the type of the heteroatoms in the hydrogen bonds, and do not further separate their types in amino acids with complex side chains, such as Asn and Gln, since these amino acids have been found to mostly form NHBs^4^. The location of the donor residues is fixed at the protein side chain. As the output, the model is expected to classify a given hydrogen bond as a SHB or a NHB.

**Figure 1:**
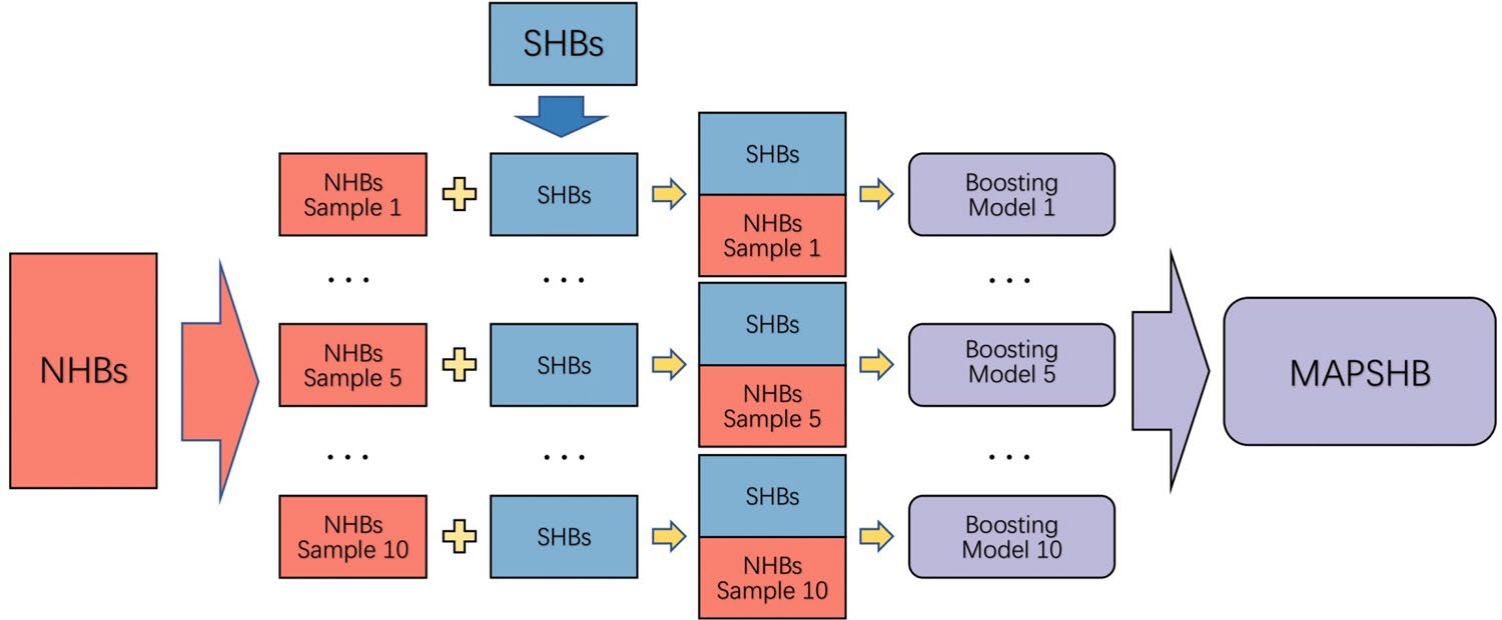
Workflow for the development of the MAPSHB model. In the first step, we randomly sample the NHBs in the training dataset and create 10 subsets that contain 6181 NHBs. In the second step, we form 10 balanced datasets by combining each subset of NHBs with all the SHBs. Next, we develop a boosting model from each of the 10 balanced training set and obtain the final MAPSHB model from their average.

We face two challenges in the development of the machine learning model. First, it is difficult to predict SHBs correctly as they occur much less frequently than NHBs in proteins. For example, SHBs take only 18.7% of our training data and a standard machine learning model would be more likely to categorize a given hydrogen bond as a NHB. Second, the model involves a considerable number of input features, each of which can take multiple values, and there are strong interaction effects among them when predicting the formation of SHBs. Taking the type of the donor residue as an example, its effect depends heavily on the type of the acceptor residue in the predictions. If Gln is the acceptor residue, the probability of observing a SHB is 32% when the donor is Thr and 11% when the donor is Lys, giving an odds ratio of 3.8 ($=(\frac{32\%}{1-32\%})/(\frac{11\%}{1-11\%}$)). In comparison, if Glu acts as the hydrogen bond acceptor, the probabilities become 65% and 23% for the donor residues Thr and Lys, respectively. In the latter case, the odds ratio of 6.2 is almost two fold of that in the former case. Therefore, it is crucial to account for the interaction effects among the 21 input features to achieve a good prediction performance of our model.

To address the first challenge, we use the undersampling strategy^45^ to develop the machine learning model. The main idea is to create multiple balanced datasets, which contain equal numbers of SHBs and NHBs, by sampling subsets of NHBs from all the training data. As SHBs and NHBs appear equally frequently in these datasets, the resulting models have enhanced performances in predicting the occurrence of SHBs and we will ensemble them to obtain the final model. To address the second challenge, we invoke the boosting model^46^ that fits a series of decision tree models sequentially and adaptively. From the tree structure of each model, we can automatically and efficiently incorporate the interaction effects among the 21 structural, chemical and sequence input features. We then combine them to form the final model, which has a significantly boosted performance in the prediction of SHBs as compared to the individual decision tree models. We have also tested other traditional models such as random forests, support vector machines and multilayer feedforward neural networks and found that these models achieve similar level results (Table S4). We have chosen the boosting based model in this work as it can provide a good interpretation to the SHB predictions. We hence take the following steps to develop the machine learning model and the workflow is schematically represented in Fig. 1.

*Step 1* Generate a subset of NHBs by randomly selecting 6181 NHBs from the entire training dataset;

*Step 2* Combine this subset of NHBs with the SHBs to form a balanced training set that contains equal numbers of SHBs and NHBs;

*Step 3* Train a boosting model on this balanced training set;

*Step 4* Repeat steps 1–3 to obtain 10 boosting models;

*Step 5* Average over the 10 models to construct the final machine learning model.

We name the resulting model as the machine learning assisted prediction of short hydrogen bonds (MAPSHB) model. We have further designed a web server for it on https://www.sas.rutgers.edu/cms/wanggroup/mapshb-model/the-mapshb-model, which allows a user to upload a protein structure and obtain the probability of each hydrogen bond as a SHB. By defining a probability threshold, we then predict a hydrogen bond to be a SHB if its probability is greater or equal to this value, or a NHB if the probability is below it. To assess the performance of the MAPSHB model, we apply it to the test dataset and examine two metrics of its predictions. One metric is precision, which is the proportion of true SHBs among all of the predicted SHBs; the other is recall, which is the proportion of the predicted SHBs among all the SHBs in the test dataset. For both metrics, larger values correspond to higher model performances.

As shown in Tables 1 and S2, one can use the probability threshold to tune the precision and recall of the MAPSHB model and modulate the predictions for a specific research need. A larger threshold value yields a prediction of fewer hydrogen bonds as SHBs, and results in a higher precision but lower recall of the model. In contrast, a smaller threshold leads to a prediction that most of the hydrogen bonds are SHBs and hence a lower precision but higher recall. From Table 1, if one requires the model to have highly precise predictions of SHBs in their protein structures, a stringent threshold of 0.996 can be used to control the precision to be 95%. However, the recall is relatively low and only 20% of SHBs in the test set are identified. If one instead wants to explore all the plausible SHBs in a protein, a small threshold of 0.062 can be chosen to reach an excellent recall of 94%. Therefore, one can use the data in Table 1 as a guidance and adjust the balance between the precision and recall of the MAPSHB model for their systems. Our recommended probability threshold is 0.870, with which the model predictions can achieve a precision of 80% while maintaining a relatively high recall of 75%. Here the best score of prediction is about 80% because we are dealing with strongly unbalanced dataset and it is difficult to reach both high precision and high recall for the model prediction.

**Table 1:** The precision and recall of the MAPSHB model with different probability thresholds. The recommended threshold and its relevant metrics are highlighted in bold.

Probability threshold	Precision (%)	Recall (%)
0.996	95	20
0.979	90	50
0.943	85	66
**0.870**	**80**	**75**
0.740	70	83
0.555	60	87
0.062	40	94

**Figure 2:**
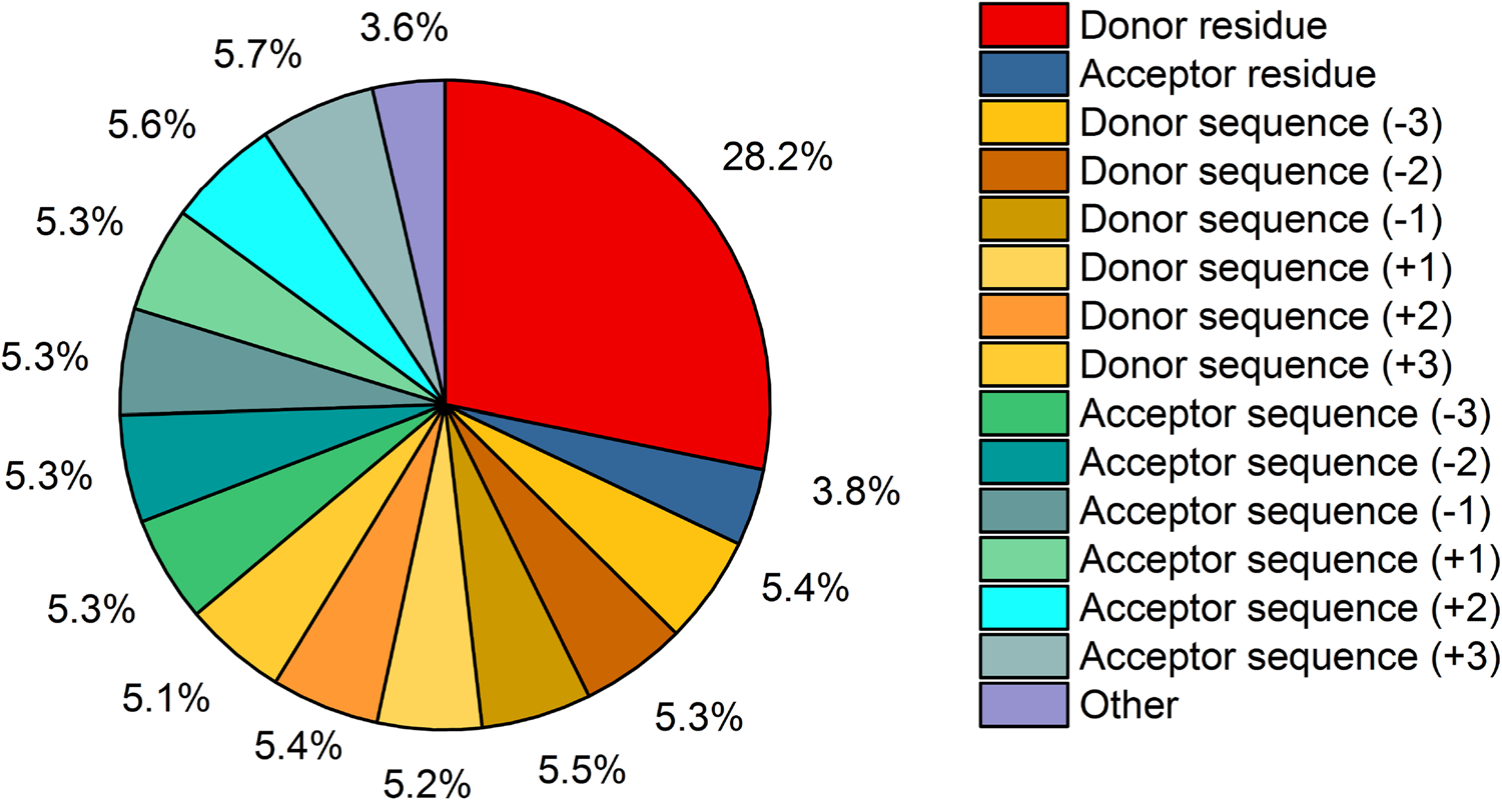
Normalized importance scores for the 21 input features of the MAPSHB model. The features that contribute $\le$1% to the predictions are grouped as the “other” type. These include the atom type, charge and secondary structure of the donor and acceptor groups and the location of the acceptor residue.

To reveal the key factors that promote the formation of SHBs between amino acids, we use the MAPSHB model and calculate the relative importance of its 21 input features^47^. As demonstrated in Fig. 2, the type of the donor residue plays a major role in determining the class of a hydrogen bond and possesses the most significant importance score of 28.2% in our predictions. From the training dataset, the donor amino acids can be divided into three groups that give vastly different probability of forming SHBs. The first group is Tyr, which has a remarkable 86% probability to form a SHB when its phenol side chain serves as the donor of a hydrogen bond. The second group includes Arg, Lys, Asn, Gln and Trp, as the probability of observing SHBs is below 15% when the N-containing functional groups of their side chains are the donors. Given their distinct preferences in forming SHBs or NHBs, the MAPSHB model can determine the class of a hydrogen bond almost solely from the donor residue when it belongs to these two groups. The third group comprises Ser, Thr and His, and their SHB-forming probabilities are 53%, 40% and 29%, respectively. This suggests that when a hydrogen bond contains the hydroxyl or imidazole side chain of these amino acids as the donor, it is feasible to form either a SHB or a NHB. In this case, the MAPSHB model cannot judge the class of the hydrogen bond only from the donor residue, and it is essential to consider the acceptor properties such as its charge, location and the residue and atom types. For example, when Thr is the donor, the probability of observing a SHB is 73.8% if the anionic Asp or Glu is the acceptor residue, and is only 29.2% if a neutral amino acid is the acceptor. Similarly, when Ser and His are the hydrogen bond donors, the presence of the anionic Asp and Glu as acceptors significantly enhances the likelihood of forming SHBs.

From Fig. 2, the amino acids next to the hydrogen bond donor and acceptor residues in the protein sequence are important features that facilitate the formation of SHBs, and each of them contributes $\sim$5% to the prediction of the MAPSHB model. Interestingly, we observe an enhanced SHB-forming probability when the donor and acceptor residues are separated by only one amino acid in the sequence, possibly because the secondary structure of the protein backbone can help position the hydrogen bonded groups in close proximity. This is particularly the case when Ser or Thr acts as the donor and Asp is the acceptor of a hydrogen bond. For instance, we observe the Ser-Xxx-Asp and Asp-Xxx-Ser (Xxx represents any amino acid) sequence patterns in 36.4% of the hydrogen bonds formed between the side chains of Ser and Asp, and they show a considerable 82.5% probability to form SHBs. As an example, we observe an Asp-Met-Ser sequence in the active site of a cyclopropanase, in which the Asp and Ser side chains form a SHB in the presence of a bound NADP$^{+}$ cofactor (PDB ID 5DP2)^48^. In Fig. 2, apart from the hydrogen bond donor and acceptor residues and their relevant sequence information, the other features such as the charge, atom type and secondary structure of the hydrogen bonded groups appear to be less important and contribute $\le$ 1% to the overall prediction. It is because they partially overlap with the residue information and only play essential roles when the hydrogen bond donor belongs to the third group of amino acids, which are associated with 25% of the data.

### Side-chain Tyr-Asp and Tyr-Glu pairs are most likely to form SHBs

Using a data-driven approach, the MAPSHB model provides useful guidelines to pinpoint SHBs in proteins. In its top 1623 predictions, the amino acid pairs have a significant (> 99%) probability of forming SHBs and 71% of them have the anionic side chains of Asp or Glu as the acceptor. Consistent with Fig. 2, the most common donors are Tyr in the first group (937 SHBs) and Ser in the third group (419 SHBs). Residues in the second group, such as Arg and Lys, are predicted to have a low probability of forming SHBs. To confirm these predictions, we further analyze all the side chain-side chain hydrogen bonds in the dataset. As shown in Table S5, the Tyr-Asp pair has the highest probability (96%) to form a SHB and this is followed by the Tyr-Glu pair (93%). The Ser-Asp and Ser-Glu pairs have a lower probability of $\sim$79% to form SHBs, but they constitute the largest portion (20%) of all the side chain-side chain SHBs. The charged Arg-Asp and Arg-Glu pairs possess strong electrostatic stabilization and form the largest amount (8671) of hydrogen bonds, but the probabilities of observing them in SHBs are below 6%. In all of the cases, the median R is about 2.6 Å for the SHBs and 2.9 Å  for the NHBs, and we do not observe significant differences between different pairs of amino acids (Table S5).

**Figure 3:**
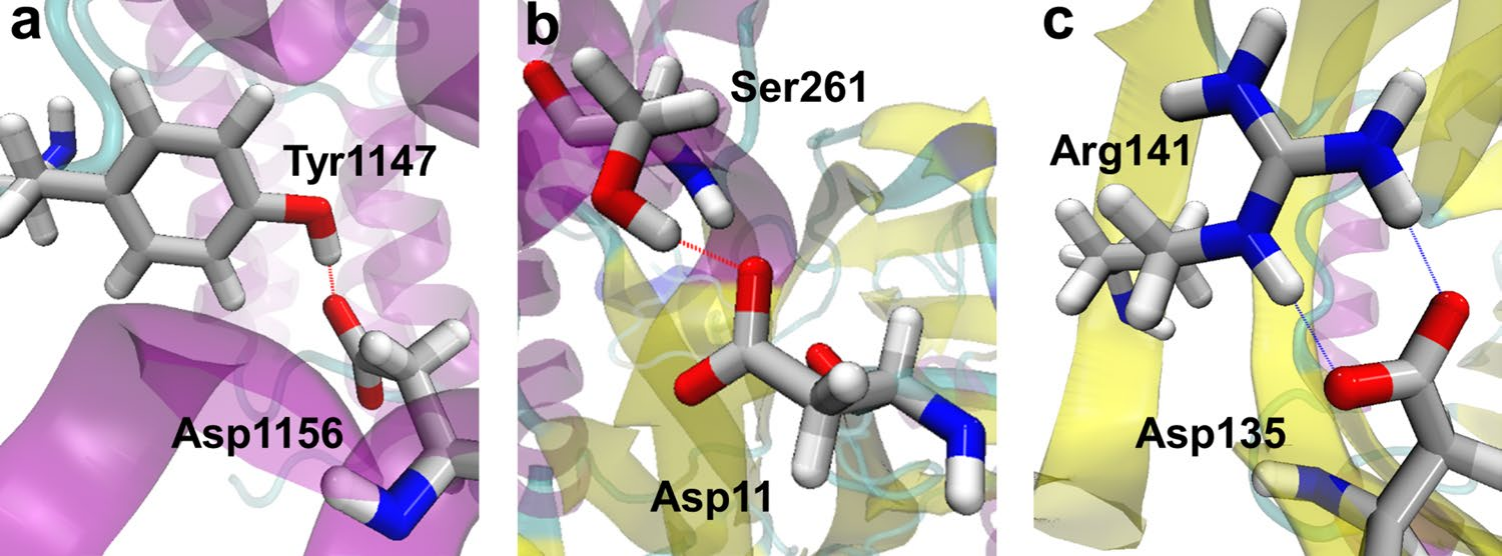
Structures of the Tyr-Asp, Ser-Asp and Arg-Asp side chain-side chain hydrogen bonds that represent their most probable geometry arrangements in proteins. (**a**) A SHB between Tyr1147 and Asp1156 (R = 2.65 Å) in a CBP bromodomain (PDB ID 5I86)^50^. (**b**) A SHB between Ser261 and Asp11 (R = 2.65 Å) in an amidase mutant (PDB ID 4LF0)^51^. (**c**) Two regular hydrogen bonds between Arg141 and Asp135 (R = 2.79 Å and 2.85 Å) in the majastridin protein (PDB ID 2NXV)^52^.

To uncover the origin of the observed trend, we choose Tyr, Ser and Arg to represent the three groups of donor amino acids and take Asp as an example acceptor residue given the structural similarities between the side chains of Asp and Glu. We have randomly selected 150 configurations for each of the Tyr-Asp, Ser-Asp and Arg-Asp side chain-side chain hydrogen bonds and performed electronic structure calculations to obtain their interaction energies. Among them, we include two representative configurations for each pair from the first and second maxima of their geometric probability distributions (Fig. S2) and evaluate how their interaction energies vary with R. As shown in Figs. 3 and S3, the Tyr-Asp and Ser-Asp pairs are highly likely to form SHBs while the Arg-Asp pair is most frequently observed as a NHB with R above 2.8 Å. In the calculation of the hydrogen bond energies, we find it crucial to account for the distinctive electrostatic environment around the donor and acceptor residues. In particular, the Tyr-Asp and Ser-Asp hydrogen bonds are often found in the protein interior (Table S6), and we implement the polarizable continuum model (PCM) with a dielectric constant of 10.0 to mimic the protein environment^49^. In contrast, the Arg-Asp hydrogen bond is mostly observed on the protein surface as the charged side chains of Arg and Asp have a significant probability of 85% and 76% to be solvent exposed, respectively (Table S6), and we use a dielectric constant of 78.4 to represent a water environment around this pair.

**Figure 4:**
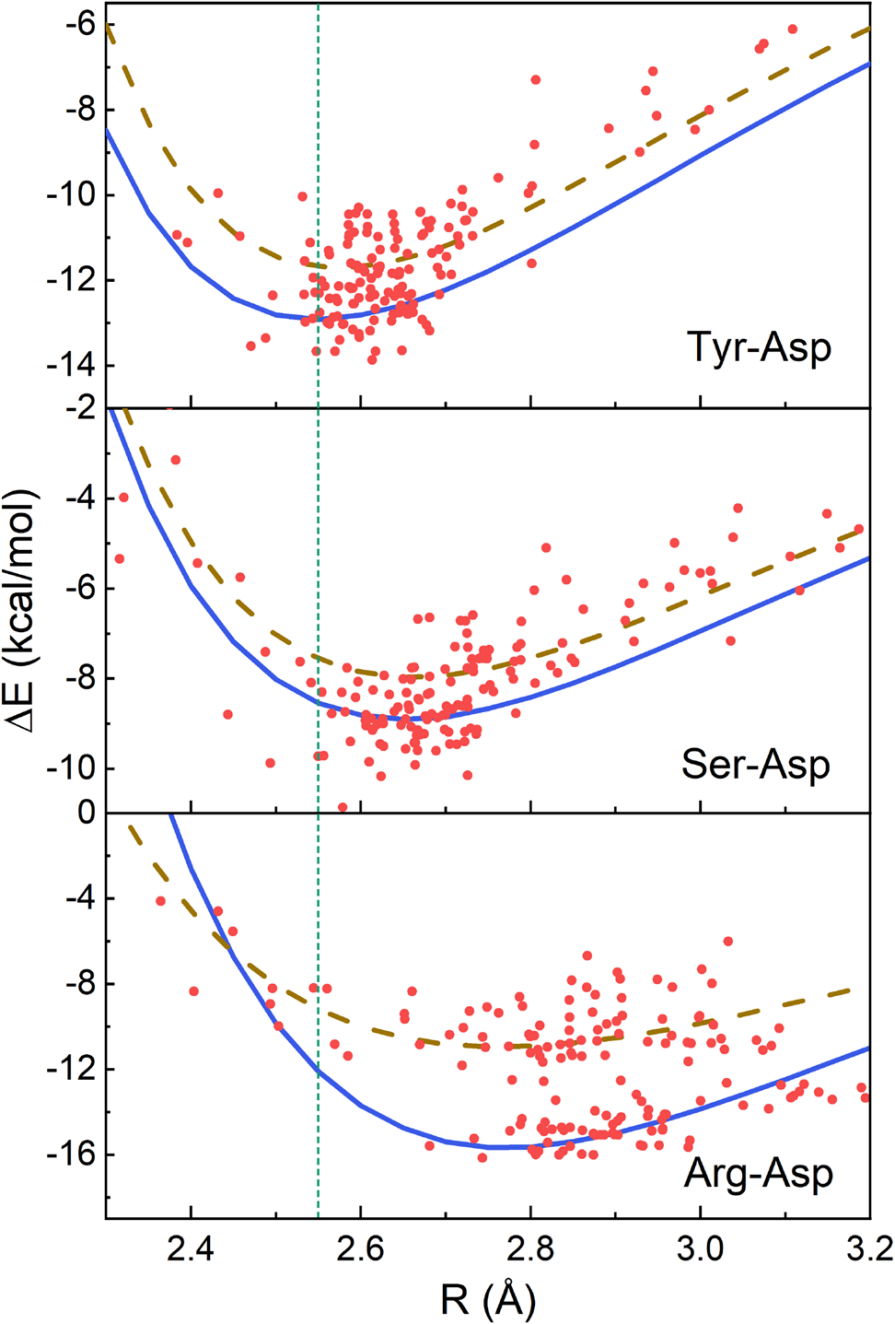
Interaction energies of the Tyr-Asp, Ser-Asp and Arg-Asp side chain-side chain hydrogen bonds. For each type of the hydrogen bond, the red dots represent the interaction energies of 150 randomly chosen configurations. We further calculate how the hydrogen bond energies of the configurations from the first and second maxima of the geometric probability distributions vary with R, which are shown as the solid and dashed lines, respectively, in each plot. The vertical line represents the potential energy minimum of the Tyr-Asp pair at R = 2.55 Å.

As shown in Fig. 4, for each type of the hydrogen bond, the interaction energies of the 150 geometry arrangements closely follow the potential energy surfaces that are computed from their most probable configurations. For example, the minima of the interaction energy occur at an O–O distance, $R_{min}$, of 2.55 Å for the Tyr-Asp hydrogen bond, and 2.65 Å for the Ser-Asp hydrogen bond. Consistent with the MAPSHB predictions, the $R_{min}$ values of both pairs are within the range of SHBs, indicating that it is energetically favorable for their donor and acceptor residues to stay in close proximity. In contrast, $R_{min}$ of the Arg-Asp hydrogen bond are at 2.75 Å in its potential energy surfaces and the interaction energies rise sharply when R shortens, confirming that this combination of amino acid side chains predominantly forms NHBs. In the following, we will analyze the most frequently observed configuration of each hydrogen bond since their potential energy curves well represent the corresponding hydrogen bond energies in proteins and are lower by 0.3–6.4 kcal/mol than those of the second most probable configurations at all hydrogen bond lengths.

**Figure 5:**
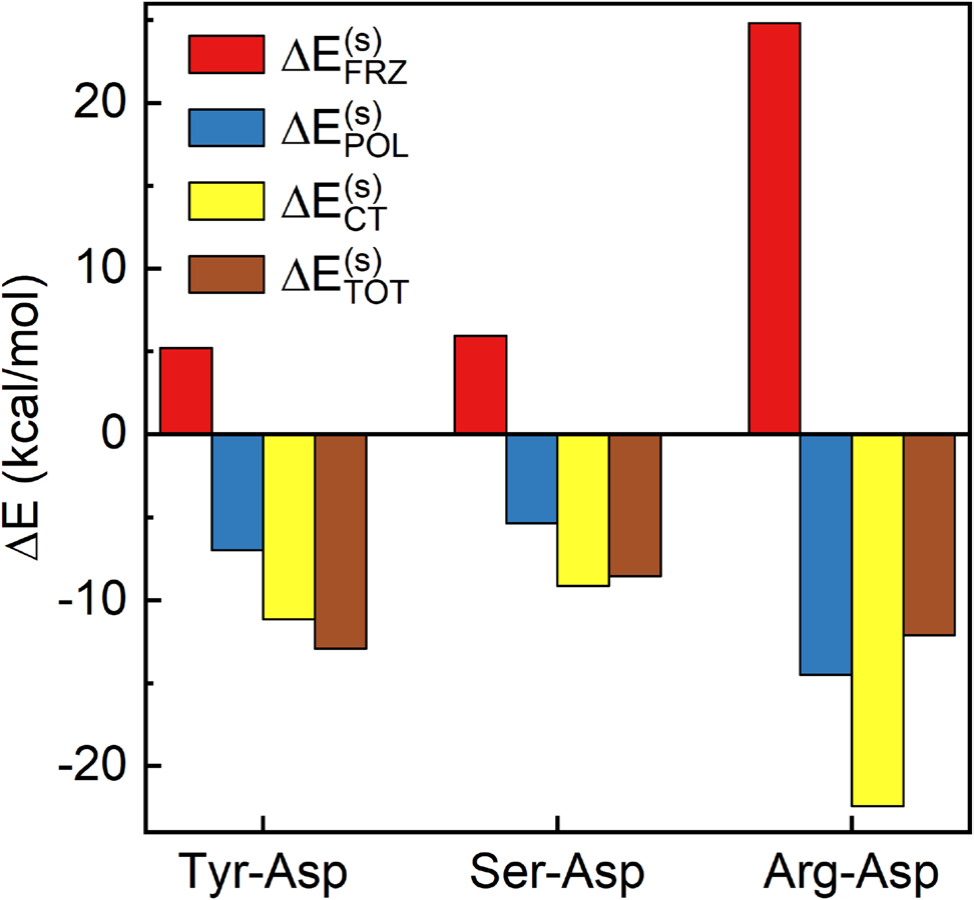
Decomposition of the interaction energies for the Tyr-Asp, Ser-Asp and Arg-Asp hydrogen bonds at R = 2.55 Å. The total interaction energy ($\Delta E_{TOT}^{(s)}$) is partitioned into the frozen interaction ($\Delta E_{FRZ}^{(s)}$), polarization ($\Delta E_{POL}^{(s)}$) and charge transfer ($\Delta E_{CT}^{(s)}$) energies.

From Fig. 4, we choose an $R_{min}$ of 2.55 Å in the potential energy curves of the Tyr-Asp, Ser-Asp and Arg-Asp pairs, and decompose their interaction energies using the ALMO-EDA(solv) method^53^ to elucidate why the Tyr-Asp combination shows the highest probability of forming SHBs in proteins. As demonstrated in Fig. 5, the total interaction energy of a hydrogen bond is partitioned into the frozen interaction, polarization and charge transfer energies^53^,

1 $$\begin{aligned} \Delta E_{TOT}^{(s)}=\Delta E_{FRZ}^{(s)}+\Delta E_{POL}^{(s)}+\Delta E_{CT}^{(s)}. \end{aligned}$$

Here the superscript (*s*) indicates that the decomposition is carried out using a solvent model. The frozen interaction energy, $\Delta E_{FRZ}^{(s)}$, describes the energy difference between a solvated hydrogen bond pair and its individually solvated, non-interacting donor and acceptor residues without any orbital relaxation. It can be further decomposed into the contributions from the permanent electrostatics, Pauli repulsion and dispersion interactions in vacuum and a solvation term (Table S7). The effects of orbital interactions and electron redistribution are incorporated in the $\Delta E_{POL}^{(s)}$ and $\Delta E_{CT}^{(s)}$ energies^53^.

From Fig. 5, the total interaction energies of the Tyr-Asp, Ser-Asp and Arg-Asp pairs are distributed among all three terms, with the frozen interaction acting to weaken the hydrogen bonds and the polarization and charge transfer interactions strengthening them. As shown in Table S7, the positive frozen interaction energies come from the large Pauli repulsion (over 55 kcal/mol), which is due to the short contact between the donor and acceptor groups, and the solvation energies (8–102 kcal/mol) as the solute-solvent interactions damp the Coulomb attraction in the hydrogen bond. In the meantime, the attractive electrostatic and dispersion interactions cancel about 90% of these unfavorable interactions, leading to a $\Delta E_{FRZ}^{(s)}$ between 5 and 25 kcal/mol for the three types of hydrogen bonds. Comparing the Tyr-Asp and Ser-Asp pairs, they share similar $\Delta E_{FRZ}^{(s)}$ values as the donor residues both contain hydroxyl groups. However, the former has longer O–H bond and more shared proton in the hydrogen bond because the phenol side chain of Tyr is more acidic (with $pK_a$ value of 10.1) than the hydroxymethyl side chain of Ser. As a result, the Tyr-Asp hydrogen bond has more prominent contributions from the polarization and charge transfer terms, which lower the interaction energy by 3.7 kcal/mol more than that of the Ser-Asp hydrogen bond. Similar behavior is observed over the whole range of R (Fig. S5), and a delicate balance of the frozen interaction, polarization and charge transfer makes the Tyr-Asp pair more stable at a short R of 2.55 Å than the Ser-Asp pair. Comparing the Tyr-Asp and Arg-Asp hydrogen bonds, the latter has significantly larger components of the intermolecular interactions because both of its donor and acceptor groups are charged (Fig. 5). For example, the polarization and charge transfer interactions in the Arg-Asp hydrogen bond give rise to a stabilization energy of -36.9 kcal/mol, which is over twice of that in the Tyr-Asp case. However, its frozen interaction is 5 times larger than that of the Tyr-Asp pair and destabilizes the overall interaction by 24.8 kcal/mol. From Table S7, this arises from a Pauli repulsion of 121.1 kcal/mol in this N–H$\cdot \cdot \cdot$O type hydrogen bond, and a solvation energy of 101.2 kcal/mol that smears out the Coulomb attraction between the cationic Arg and anionic Asp residues. From Fig. S5, the repulsive frozen interaction of the Arg-Asp pair decays rapidly when R lengthens, making it more stable when the donor and acceptor residues are separated in the NHB range.

Given their prevalence in proteins, we have considered the hydrogen bonds that form between the hydroxyl side chains of Tyr, Ser or the guanidinium side chain of Arg and the carboxylate side chains of Asp or Glu, and identified that their preferred lengths come from a balance of several competing effects. As demonstrated in Fig. 5, the frozen interaction tends to weaken a SHB mainly because the large Pauli repulsion prevents the donor and acceptor residues from staying in close proximity. The solute-water interactions also considerably destabilize hydrogen bonds that contain charged residues, and are particularly repulsive when a hydrogen bond forms between cationic and anionic residues, as in the cases of the Arg-Asp and Lys-Asp pairs. While electrostatic interactions play an important role in counteracting these effects, polarization and other purely quantum mechanical effects such as dispersion and charge transfer also contribute 20–28% to the overall stabilization energy (Table S7). Among the neutral amino acids, Tyr has the most ionizable side chain due to its conjugated ring structure and hence is capable of sharing its proton with the Asp/Glu acceptor residue. As a result, the stabilization effects from polarization and charge transfer dominate in the Tyr-Asp hydrogen bond and make it energetically most favorable at a short heteroatom separation of 2.55 Å. Furthermore, the interaction energy of the Tyr-Asp pair is mostly invariant with the solvation condition, whereas the aqueous environment considerable weakens the charged Arg-Asp hydrogen bond (Fig. S4) and makes it most stable with R above 2.7 Å. Due to the interplay of the frozen interaction, polarization and charge transfer interactions, the Tyr-Asp/Glu pair has the highest probability of forming SHBs among the commonly observed combinations of amino acid side chains.

## Conclusions

In this work, we have developed a MAPSHB model that effectively predicts SHBs in proteins that form between amino acids and have the side chain of an amino acid as the donor residue. By tuning the probability threshold, we successfully control the precision and recall of its predictions to both around 80%. We have designed a web server for the MAPSHB model on https://www.sas.rutgers.edu/cms/wanggroup/mapshb-model/the-mapshb-model, which allows a user to input a protein structure with low to moderate resolution and identify probable SHBs in the biomolecule. It will provide additional restraints for the experimental and computational refinement of protein structures, and facilitate the determination of the structure and functional roles of SHBs in proteins.

From the MAPSHB model, we uncover three main features that promote the formation of SHBs in proteins. First, the donor amino acid plays a major role in determining the class of a hydrogen bond. This is particularly the case for Tyr, which exhibits a strong preference for the formation of SHBs. Second, the carboxylate side chains of Asp and Glu are the most frequently observed acceptors of SHBs in proteins. They strongly modulate the likelihood of observing these compact structures when the donor residue belongs to the third group of amino acids, namely Ser, Thr and His. Finally, the sequence of amino acids next to the hydrogen bond groups can also facilitate the formation of these close contacts. For example, there is an enhanced probability of observing a close contact in a Ser-Asp or Thr-Asp hydrogen bond when the donor and acceptor residues are separated by only one amino acid in the sequence. Following the first two rules, we find that the Tyr-Asp and Tyr-Glu side chain-side chain hydrogen bonds have the highest probability of forming a SHB in proteins. Combining electronic structure calculations and energy decomposition analysis, we compare the Tyr-Asp pair with the Ser-Asp and Arg-Asp hydrogen bonds and reveal that this trend comes from a competition of intermolecular interactions. While the frozen interaction tends to push the heteroatoms away in space, Coulomb attraction, polarization and other purely quantum mechanical effects such as dispersion and charge transfer play key roles in stabilizing a SHB. A delicate balance of these effects make the Tyr-Asp and Ser-Asp pairs energetically favorable at an O-O distance below 2.7 Å. In comparison, while the cationic Arg and anionic Asp tend to form the largest amount of hydrogen bonds, their interactions with the aqueous solution make the Arg-Asp pair most stable as a NHB. The predictions of the MAPSHB model, in conjunction with our elucidation of the origin of these hydrogen bonding interactions, will guide the design of novel protein systems that take advantage of SHBs to enhance their functions in biochemical and materials applications.

## Methods

### Data preparation for the machine learning model

We obtained 2171 high-resolution protein structures from the PDB, and removed the crystallographic waters. Hydrogen atoms were added using the Amber 2016 package^54^. We then used the Amber14SB force field^55^ to model the proteins and the general Amber force field^56^ to describe the ligands, and optimized the protein structures by keeping all the non-hydrogen atoms fixed in space. The cpptraj program in AmberTools16^54^ was used to identify the hydrogen bonds and the secondary structures that the donor and acceptor residues were in.

### Development of the machine learning model

We used the generalized boosted regression modeling (gbm) function^57^ in the R programming language to develop a boosting model for each balanced dataset (step 3 in our procedure). We set the shrinkage as 0.01 and the number of decision tree models as 5000. The interaction depth was treated as a tuning parameter, for which we used a 10-fold cross validation to choose the optimal value. Here we randomly split a balanced dataset into 10 groups with equal sizes, and for each group, we trained the model on the remaining 9 groups and recorded the validation error on this group. We then calculated the average validation error of all the groups for each interaction depth. By varying the interaction depth from 1 to 15, we determined the optimal value that minimized the average validation error. Using the optimal interaction depth, we refitted the boosting model on the whole balanced dataset and saved it as the final boosting model. For each boosting model, we used the varImp function in the caret package^58^ to calculate the importance score of each feature. The final importance scores for the MAPSHB model were calculated from averaging over the 10 boosting models.

### Electronic structure calculations

We randomly chose 150 configurations for each of the Tyr-Asp, Ser-Asp and Arg-Asp side chain-side chain hydrogen bonds from the dataset and ensured that their R covered the whole range between 2.3 and 3.2 Å. For each pair, we performed electronic structure calculations to obtain its interaction energy from the energy difference between the hydrogen bonded dimer and the corresponding monomers, $\Delta E=E_{Hbond}-(E_{Donor}+E_{Acceptor})$. We also considered two configurations for each pair at the first and second maxima of their geometric probability distributions (Fig. S2), and computed their hydrogen bond energies at different R by keeping the relative orientation of the hydrogen bond donor and acceptor groups and changing R of the hydrogen bond. These hydrogen bond configurations are shown in Figs. 3 and S3. In all the calculations, the side chains of Tyr, Ser, Arg and Asp were represented using the compounds 4-ethylphenol, ethanol, protonated 2-butylguanidine and propanoate, respectively, and the interaction energies were computed after optimizing the geometry of each structure with the non-hydrogen atoms fixed in space. The electronic structure was described using the B3LYP density functional^59^, the D3(op) dispersion correction^60^ and the aug-cc-pVDZ basis set. Here density functional theory, rather than post-Hartree-Fock methods such as MP2 and coupled cluster, was used to enable the energy decomposition analysis with the continuum solvation model. The conductor-like PCM method was used to mimic the protein and solvent environment. A dielectric constant of 10.0 was used for the Tyr-Asp and Ser-Asp pairs^61^, and a value of 78.4 was used for the Arg-Asp pair based on the relative solvent accessibility of the amino acids (more discussions are provided in the Supplementary Information). The resulting interaction energies of the hydrogen bonds are shown in Fig. 4. For the energy decomposition analysis in Fig. 5, we used the ALMO-EDA(solv) method^53^ as implemented in the Q-Chem 5.3 software^62^.

## Supplementary Information

Supplementary Information.

## Data Availability

All data generated or analysed during this study are included in this published article and its Supplementary Information file.
