# Supplementary material for: Effective prediction of short hydrogen bonds in proteins via machine learning method
Source: Sci Rep. 2022 Jan 10;12:469. doi: 10.1038/s41598-021-04306-4 (PMC8748993; doi:10.1038/s41598-021-04306-4)
Supplement: Supplementary file 1 — Supplementary Information. [file 41598_2021_4306_MOESM1_ESM.pdf]

Supplementary Information

Effective prediction of short hydrogen bonds in proteins via machine learning method

Shengmin Zhou[1,+], Yuanhao Liu[2,+], Sijian Wang[2], and Lu Wang[1,*]

[1]Department of Chemistry and Chemical Biology, Institute for Quantitative Biomedicine, Rutgers University, Piscataway, NJ 08854, USA; [2]Department of Statistics, Institute for Quantitative Biomedicine, Rutgers University, Piscataway, NJ 08854, USA

[+]S.Z. and Y.L. contributed equally to this work.

[*]To whom correspondence should be addressed: lwang@chem.rutgers.edu

1. Supplementary methods

1.1 Preparation of the hydrogen bond dataset

After obtaining 2171 high-resolution structures from the PDB, we found that some proteins appeared multiple times in the dataset because researchers determined the structures of the wild-type and mutated proteins as well as those after protein splicing or ligand binding. Considering their structural similarity, we removed the "redundant" structures with the same protein name and only included one structure with the highest resolution for each peptide and protein in the training dataset. To further assess the redundancy of the peptide and protein sequences, we used the CD-HIT web server[1] to analyze the resulting 782 structures in the training set. After setting the sequence identity cutoff to 0.9, we found that the 853 chains reduced to 741 independent chains, suggesting that there was still a small degree of redundancy in the training data. However, we expect this small sequence redundancy to have minor impact on the development of the machine learning model. In comparison, we kept all the structures in the test set so that the model predictions covered all the available data. From the resulting 1260 peptide and protein structures in the training and test datasets, we collected all the hydrogen bonds that formed from two amino acids and had the side chain of an amino acid as the donor group using the Amber 2016 software package.[2]

We obtained 21 structural, chemical and sequence features for each hydrogen bond, including the residue and atom types, charge and location of the hydrogen bond donor and acceptor and the relevant secondary structures and sequence information. Specifically, we used the DSSP algorithm[3] as implemented in Amber 2016[2] to determine the secondary structure that a residue resided in. We then gathered the sequence within 3 amino acids of the donor and acceptor residues of a hydrogen bond, which we referred to as their +3/-3 residues. We found that some hydrogen bonds were near the C- or N-terminus of a peptide or protein and missed one or more of the +3/-3 residues around their donor and acceptor groups. In these cases, we marked the missing residues as XXX. In addition, some proteins had local structural uncertainty with multiple possible amino acids in one position, and we marked these uncertain residues as +++ in the sequence analysis. The probabilities of observing the XXX and +++ residues were 5.1% and 0.1%, respectively, in the entire dataset. Given their rare occurrence, we removed all the hydrogen bonds with the XXX or +++ residues in their +3/-3 sequences in both the training and test datasets.

We found that the location of the donor and acceptor residues can play an important role in determining the class of a hydrogen bond. As shown in Table S1, the probability of observing a hydrogen bond to form a SHB is below 1.5% when the donor residue is in the protein backbone. In comparison, this probability can go up to 24% when the donor residue is in the side chain of an amino acid. To avoid highly imbalanced data, we only included the hydrogen bonds whose donor residues are in the protein side chains in the training and test datasets.

1.2 Development of the machine learning model

The machine learning model is expected to predict whether a hydrogen bond that forms between amino acids is a SHB or a NHB. This can be viewed as a classification problem, for which a number of suitable machine learning approaches are available in the literature. However, since the training dataset of the hydrogen bonds are imbalanced with the number of SHBs much smaller than that of NHBs (Table S1), a standard model would provide strongly biased predictions that favor NHBs. As an example, if the model simply classifies all the hydrogen bonds as NHBs, the accuracy of the prediction (on the test dataset) would be as high as 81.8%, although these predictions are clearly incorrect. As discussed in the main text, we used the undersampling method[4] to handle the imbalanced training dataset.

We used the boosting method to develop the machine learning model. A step-by-step procedure of the method is shown in Algorithm 1.[5] We denote the training dataset, which contains N hydrogen bonds, as $(\mathbf{x}_1, y_1), \cdots, (\mathbf{x}_N, y_N)$. For the nth hydrogen bond, $\mathbf{x}_n$ represents its 21 structural, chemical and sequence features, and y_n represents its class with a value of 1 for a SHB and -1 for a NHB. In this model, we want to determine a function $p(\mathbf{x})$ that predicts the probability of a hydrogen bond with the parameters $\mathbf{x}$ to form a SHB based on the training data. The boosting model ensembles M decision tree models $f_m(\mathbf{x})$ to form a strong model $f(\mathbf{x})$,[5] which is a transformation of $p(\mathbf{x})$ and can be used to determine the class of a given hydrogen bond. An example decision tree model is shown in Fig. S1, from which we classify a given hydrogen bond by checking each condition of the judgement of the tree. The boosting method is adaptive as these tree models are subsequently trained using a weighted form of the hydrogen bond dataset, and the weighting coefficient ω_n associated with each data point depends on the performance of the previous model.

Algorithm 1 The boosting method

Inputs: $(\mathbf{x}_1, y_1) \cdots, (\mathbf{x}_N, y_N)$
Step 1: Initialize the coefficients of data weighting $\{w_n\}$ by setting $w_n^{(1)} = \frac{1}{N}, n = 1, \cdots, N$;
Step 2:
for $m = 1, \cdots, M$ **do**
(a) Fit a tree model $f_m(\mathbf{x})$ to the training data by minimizing the weighted error function

$$J_m = \sum_{n=1}^{N} w_n^{(m)} I\left(f_m(\mathbf{x}) \neq y_n\right),$$

where $I\left(I\left(f_m(\mathbf{x}) \neq y_n\right)\right)$ is the indicator function. It equals 1 when $f_m(\mathbf{x}) \neq y_n$, and equals 0 otherwise.
(b) Evaluate the quantity

$$\epsilon_m = \frac{\sum_{n=1}^{N} w_n^{(m)} I\left(f_m(\mathbf{x}) \neq y_n\right)}{\sum_{n=1}^{N} w_n^{(m)}}$$

and use this quantity to evaluate

$$\alpha_m = \ln\left\{\frac{1-\epsilon_m}{\epsilon_m}\right\}$$

(c) Update the data weighting coefficients

$$w_n^{(m+1)} = w_n^{(m)} \exp\left\{\alpha_m \left(f_m(\mathbf{x}) \neq y_n\right)\right\}$$

Step 3: Make predictions using the final model

$$f(\mathbf{x}) = \sum_{m=1}^{M} \alpha_m f_m(\mathbf{x}))$$

and the predicted probability that $\mathbf{x}$ belongs to the SHB class is

$$p(\mathbf{x}) = \frac{1}{1+e^{-2f(\mathbf{x})}}$$

After developing the MAPSHB model, we applied it to the test dataset and calculated its confusion matrix. The results with different threshold probabilities are shown in Table S2. Here the MAPSHB model was trained on all the hydrogen bonds whose donor residues were on the protein side chain, regardless of the location of the acceptor residues. From Table S1, the location of the donor residue plays an important role in determining the class of a hydrogen bond. Considering that the same rule might apply to the type of the acceptor residue, we further split the training dataset based on the position of the acceptor residues (backbone or side chain of a protein) and repeated the training process to develop two new machine learning models. The confusion matrices for the two models are shown in Table S3. We then combined the predicted results of these two models and compared them with those predicted from the MAPSHB model. All the predictions were made with a probability threshold of 0.5. We found no significant difference between the last two rows of Table S3, validating our choice of the training dataset for the MAPSHB model.

In addition to the boosting model, we also tested a few other commonly used machine learning models, including the support vector machine (SVM), random forest and multilayer feedforward neural network models. All models were developed using the undersampling strategy. The precision and recall of their predictions as applied to the test dataset are shown in Table S4. The boosting, SVM and neural network models have similar performances in their precision and recall. However, the precision of the predictions from the random forest model is considerably lower than the others and thus we do not consider this method in this work. Compared with the SVM and neural network models, the boosting model is based on decision trees and can provide a better interpretation to the SHB predictions, which makes it our model of choice.

1.3 Calculations of the solvent-accessible surface areas of amino acids

The chemical properties of biological hydrogen bonds depend strongly on their solvation environment. As the 20 common amino acids have different polarity and hydrophobicity, hydrogen bonds formed from them can be located at different places in proteins. For example, amino acids with polar or charged side chains are more likely to be on the protein surface and exposed to the aqueous environment, while those with non-polar side chains are often buried inside the protein. From the training and test datasets, we summarized the occurrence and median lengths of hydrogen bonds formed from the side chains of amino acids in Table S5. To estimate the solvation condition of these biological hydrogen bonds, we first calculated the solvent-accessible surface area (SASA) of the relevant amino acids in the peptides and proteins, which represented the surface areas of these residues accessible to the solvent water molecules. The calculations were carried out with the FreeSASA software package[6] using Lee and Richards' algorithm[7] and a probe radius of 1.4 Å. Next, we computed the relative solvent accessibility (RSA)

values, which accounted for the size differences of the amino acids and allowed us to compare the solvent accessibility among different amino acids. RSA was defined as the SASA of a residue normalized by a maximum possible value of the residue,

$$RSA = \frac{SASA}{SASA_{max}}. \quad [1]$$

The theoretical maximum SASA values from the study of Tien and coworkers were taken as the $SASA_{max}$ values.[8]

For each type of the side chain-side chain hydrogen bonds, we calculated the average RSA values of its donor and acceptor residues from the entire dataset and listed the results in Table S6. Considering that a residue is usually defined as surface exposed if its RSA is $\geq 5\%$,[9–12] we also included the probability of finding a hydrogen bonded residue on the surface of the peptide or protein, P_s, in Table S6. Here for a specific type of hydrogen bond, if its donor or acceptor appeared N times in the hydrogen bond dataset with n cases where the corresponding RSA value was $\geq 5\%$, we computed P_s as

$$P_s = \frac{n}{N}. \quad [2]$$

1.4 Geometries and potential energy surfaces of the Tyr-Asp/Glu, Ser-Asp/Glu and Arg-Asp/Glu hydrogen bonds

When the side chains of Tyr, Ser and Arg form hydrogen bonds with the carboxylate side chains of Asp and Glu, one O atom of the carboxylate group serves as the hydrogen bond acceptor while the other can play an important role in the cooperativity effects of proteins.[13] The energetics of the hydrogen bonds may also change as the two O atoms take different orientations. We schematically represent such a side chain-side chain hydrogen bond, D–H· · ·A, below.

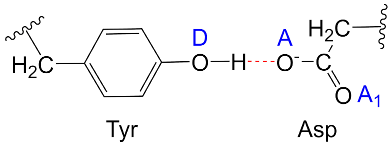

We defined the $D - A - A_1$ angle as θ and used it to represent the orientations of the two O atoms in the carboxylate side chains of Asp and Glu. From 14449 Tyr-Asp/Glu, Ser-Asp/Glu or Arg-Asp/Glu side chain-side chain pairs in the training and test datasets, we calculated the probability for a hydrogen bond to be at length R and angle θ, $P(R, \theta)$, and plotted the distributions of $P(R, \theta)$ in Fig. S2. For the same donor residue, the distributions of $P(R, \theta)$ are similar when the acceptor residue is Asp or Glu, so we will use Asp as the example in the following analyses. From Fig. S2a-c, the most commonly observed configuration of the Tyr-Asp hydrogen bond has R = 2.65 Å and $\theta = 150°$. Its probability distribution also has a second peak at R = 2.60 Å and $\theta = 100°$. In comparison, the most probable configuration of the Ser-Asp hydrogen bond has R = 2.65 Å and $\theta = 90°$, with a second maximum at R = 2.70 Å and $\theta = 150°$. While the most favored configurations of the Tyr-Asp and Ser-Asp pairs both form SHBs, their θ values differ by 60°, corresponding to two different relative positions of the A1 atom in the hydrogen bond, as demonstrated below. For the Arg-Asp pair, the most frequently observed configuration has R = 2.85 Å and $\theta = 90°$, and a second peak occurs at R = 2.85 Å and $\theta = 150°$ (Fig. S2). In both cases, the Arg-Asp side chain-side chain pairs form NHBs.

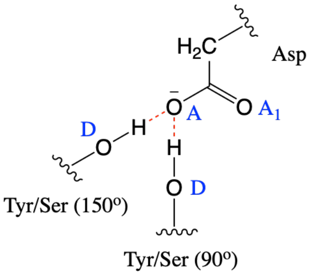

1.5 Calculations of the hydrogen bond energies

For each of the Tyr-Asp, Ser-Asp and Arg-Asp side chain-side chain hydrogen bonds, we obtained a representative configuration within a region of -0.05 ∼ +0.05 Å in R and -10 ° ∼ +10° in θ around its two peaks of the geometric probability distributions (Fig. S2). The configurations are shown in Figs. 3 and S3. We then represented the side chains of Tyr, Ser, Arg and Asp as 4-ethylphenol, ethanol, protonated 2-butylguanidine and propanoate, respectively, and calculated the interaction energy of the hydrogen bonds as a function of R using the B3LYP density functional,[14] the D3(op) dispersion correction[15] and the aug-cc-pVDZ basis set. Specifically, for a hydrogen bond pair, we scanned its R by translating the acceptor group while maintaining the relative orientation of the donor and acceptor groups, and optimizing the resulting structure with the non-hydrogen atoms fixed in space. We applied the polarizable continuum model (PCM) in the electronic structure calculations to approximately account for the condensed-phase environment around the hydrogen bond donor and acceptor residues. From Table S6, the Tyr-Asp and Ser-Asp hydrogen bonds are likely to form in the inside of a protein, while the Arg-Asp hydrogen

bonds are mostly exposed to the solvent environment. We thus used a dielectric constant, ε, of 10.0 for the Tyr-Asp and Ser-Asp pairs to mimic a protein environment,[16–18] and an ε of 78.4 for the Arg-Asp hydrogen bond to represent its surrounding aqueous environment. The resulting potential energy surfaces of the hydrogen bond interactions are shown in Fig. S4 (red solid and dashed lines). For the Tyr-Asp and Ser-Asp pairs, the potential energy surfaces for the two configurations are very similar with the most probable configurations having lower energies at all R. For the Arg-Asp pair, both configurations are most stable when they form NHBs. However, the most probable configuration has much lower interaction energies compared to the second most probable structure, consistent with the observation in Fig. S2 that an R of 2.85 Å and an angle of 90° are more preferred in the formation of Arg-Asp hydrogen bonds.

To examine the impact of our choice of ε in the solvent model, we repeated the calculations of the hydrogen bond energies using $\varepsilon = 78.4$ for the Tyr-Asp and Ser-Asp hydrogen bonds, and $\varepsilon = 10.0$ for the Arg-Asp hydrogen bonds. As demonstrated in Fig. S4, the potential energy curves for the Tyr-Asp and Ser-Asp pairs resemble each other when different dielectric constants are used, with an energy difference < 2 kcal/mol. This means that the hydrogen bond energies are nearly invariant with the ε values used in the PCM calculations. In contrast, the energies of the Arg-Asp hydrogen bond are very sensitive to the solvent environment as the energy can differ by 10.5 kcal/mol when different ε values are used.

2. Supplementary tables

Table S1. Summary statistics of the training and test datasets when the donor (D) and acceptor (A) residues of a hydrogen bond is in the backbone (B) or side chain (S) of a peptide or protein. Given the low probability of observing a SHB when the donor residue is in the backbone, in this work, we only consider the SHBs and NHBs whose donor groups are in the protein side chains.

Location Combination (DA)	Number of SHBs	Number of NHBs	Percentage of SHBs
SS	6711	21619	23.7%
SB	3450	23252	12.9%
BS	347	25095	1.4%
BB	375	149991	0.2%
SS + SB	10161	44871	18.5%

Table S2. The confusion matrices of the MAPSHB model with different probability thresholds. The results are obtained by applying the MAPSHB model to the test dataset. The number of true or false SHBs means the number of SHBs or NHBs that are predicted as SHBs, respectively. The Number of true or false NHBs is the number of NHBs or SHBs that are predicted as NHBs, respectively.

Probability Threshold	Number of True SHBs	Number of False SHBs	Number of True NHBs	Number of False NHBs
0.996	780	41	17901	3200
0.979	2005	218	17724	1975
0.943	2628	462	17480	1352
0.870	3004	751	17191	976
0.740	3301	1414	16528	679
0.555	3482	2316	15626	498
0.062	3758	5633	12309	222

Table S3. The confusion matrices for two machine learning models that are trained with the hydrogen bonds whose donor residues are in the side chain (S) of a protein and the acceptor residues are in the backbone (B) or side chain (S) of the protein. The prediction results of the MAPSHB model are also included. The probability thresholds for all the models are set to be 0.5. The number of true or false SHBs means the number of SHBs or NHBs that are predicted as SHBs, respectively. The number of true or false NHBs is the number of NHBs or SHBs that are predicted as NHBs, respectively. The results from the two models (SS+SB) are very similar to those from the MAPSHB model.

Location (DA)	Number of True SHBs	Number of False SHBs	Number of True NHBs	Number of False NHBs
SS	2215	1059	7373	349
SB	1264	1395	8115	152
SS + SB	3479	2454	15488	501
MAPSHB Prediction	3526	2595	15347	454

Table S4. The evaluation metrics for different machine learning models. The probability thresholds for all the models are set to be 0.5.

Models	Precision	Recall
Boosting	57.6%	88.6%
Support Vector Machine	60.8%	88.2%
Random Forest	49.2%	91.5%
Neural Network	59.2%	87.5%

Table S5. Occurrence of the side chain-side chain hydrogen bonds in the entire data set. For each amino acid pair, the numbers and median lengths of SHBs and NHBs and the probability of forming a SHB are also listed. The table is ordered by the number of hydrogen bonds that each pair forms.

Donor	Acceptor	Number of SHBs+NHBs	Number of SHBs	Number of NHBs	Median R of SHBs (Å)	Median R of NHBs (Å)	P_{SHBs}
Arg	Asp	4654	179	4475	2.64	2.91	3.85%
Arg	Glu	4017	230	3787	2.63	2.91	5.73%
Lys	Asp	1191	198	993	2.66	2.89	16.62%
Ser	Asp	1159	920	239	2.65	2.93	79.38%
Lys	Glu	1122	251	871	2.64	2.90	22.37%
Thr	Asp	1113	785	328	2.65	2.90	70.53%
Tyr	Asp	821	786	35	2.62	2.90	95.74%
Tyr	Glu	710	661	49	2.62	2.94	93.10%
Asn	Asp	707	9	698	2.59	2.93	1.27%
Gln	Asp	679	14	665	2.59	2.93	2.06%
Ser	Glu	572	443	129	2.65	2.87	77.45%
His	Asp	549	274	275	2.66	2.87	49.91%
Arg	Asn	526	12	514	2.64	2.92	2.28%
Asn	Asn	504	6	498	2.60	2.94	1.19%
Arg	Gln	480	23	457	2.67	2.91	4.79%
Asn	Glu	466	10	456	2.57	2.93	2.15%
Gln	Glu	438	14	424	2.60	2.95	3.20%
Arg	Ser	430	5	425	2.67	2.95	1.16%
Thr	Glu	424	349	75	2.63	2.88	82.31%
Arg	Thr	414	3	411	2.65	2.93	0.72%
His	Glu	356	172	184	2.65	2.86	48.31%
Arg	Tyr	351	4	347	2.69	2.94	1.14%
Gln	Asn	340	8	332	2.64	2.94	2.35%
Asn	Thr	281	4	277	2.62	2.96	1.42%
Asn	Ser	275	5	270	2.66	2.93	1.82%
Asn	Gln	273	4	269	2.58	2.92	1.47%
Thr	Asn	263	121	142	2.67	2.89	46.01%
Trp	Asp	238	2	236	2.69	2.90	0.84%
Gln	Ser	224	0	224	/	2.97	0.00%
Thr	Thr	223	84	139	2.67	2.87	37.67%
Lys	Asn	217	38	179	2.66	2.92	17.51%
Gln	Gln	212	7	205	2.64	2.93	3.30%
Gln	Thr	211	0	211	/	2.99	0.00%
Trp	Glu	209	2	207	2.64	2.88	0.96%
Ser	Asn	207	114	93	2.64	2.89	55.07%
Thr	Ser	194	59	135	2.67	2.90	30.41%
Lys	Ser	168	11	157	2.67	2.90	6.55%
Lys	Gln	167	31	136	2.65	2.92	18.56%
Ser	Ser	158	51	107	2.67	2.95	32.28%
Lys	Thr	147	12	135	2.66	2.90	8.16%
Ser	Thr	139	47	92	2.67	2.94	33.81%
Asn	Tyr	131	0	131	/	3.00	0.00%
Gln	Tyr	122	4	118	2.56	2.98	3.28%
Ser	His	117	36	81	2.68	2.89	30.77%
Tyr	Asn	111	97	14	2.64	2.95	87.39%
Thr	His	108	27	81	2.68	2.86	25.00%

Donor	Acceptor	Number of SHBs+NHBs	Number of SHBs	Number of NHBs	Median R of SHBs (Å)	Median R of NHBs (Å)	P_{SHBs}
Tyr	His	106	70	36	2.66	2.88	66.04%
Trp	Thr	104	0	104	/	2.91	0.00%
Trp	Gln	97	0	97	/	2.88	0.00%
Tyr	Gln	94	81	13	2.67	2.91	86.17%
Thr	Gln	93	54	39	2.66	2.85	58.06%
His	Thr	91	27	64	2.66	2.91	29.67%
Ser	Gln	88	49	39	2.65	2.90	55.68%
Tyr	Thr	87	52	35	2.66	2.91	59.77%
His	Tyr	81	74	7	2.65	2.86	91.36%
Trp	Tyr	81	0	81	/	2.99	0.00%
Lys	His	79	1	78	2.69	2.91	1.27%
Arg	His	72	0	72	/	2.98	0.00%
His	Ser	71	26	45	2.67	2.88	36.62%
Trp	Ser	71	0	71	/	2.90	0.00%
Tyr	Ser	71	46	25	2.67	2.85	64.79%
Lys	Tyr	70	8	62	2.65	2.97	11.43%
His	Asn	64	17	47	2.67	2.87	26.56%
Tyr	Tyr	62	43	19	2.65	2.84	69.35%
Thr	Tyr	61	17	44	2.68	2.88	27.87%
His	His	59	3	56	2.70	2.89	5.08%
Trp	Asn	54	0	54	/	2.89	0.00%
His	Gln	52	20	32	2.67	2.84	38.46%
Asn	His	44	0	44	/	3.02	0.00%
Gln	His	31	0	31	/	2.98	0.00%
Trp	His	29	0	29	/	2.95	0.00%
Ser	Trp	29	0	29	/	3.07	0.00%
Ser	Tyr	28	9	19	2.67	2.96	32.14%
Arg	Arg	13	1	12	2.70	3.08	7.69%
Gln	Arg	6	0	6	/	3.02	0.00%
Lys	Arg	6	1	5	2.70	2.97	16.67%
Lys	Trp	4	0	4	/	3.06	0.00%
Asn	Arg	3	0	3	/	3.11	0.00%
Ser	Arg	2	0	2	/	3.03	0.00%
Thr	Trp	2	0	2	/	3.11	0.00%
Asn	Lys	1	0	1	/	3.20	0.00%
His	Lys	1	0	1	/	3.07	0.00%
Lys	Lys	1	0	1	/	2.95	0.00%
Arg	Trp	1	0	1	/	3.19	0.00%
Asn	Trp	1	0	1	/	3.18	0.00%
Gln	Trp	1	0	1	/	2.97	0.00%
Tyr	Trp	1	0	1	/	3.05	0.00%

Table S6. Average RSA and probability of surface exposure of the side chain-side chain hydrogen bonds. The hydrogen bond pairs are arranged in the same order as in Table S5.

Donor	Acceptor	RSA_{donor}	$RSA_{acceptor}$	$P_{s,donor}$	$P_{s,acceptor}$
Arg	Asp	0.2287	0.1935	84.62%	75.87%
Arg	Glu	0.2275	0.1970	82.72%	76.75%
Lys	Asp	0.2958	0.2369	88.50%	84.38%
Ser	Asp	0.2138	0.2007	68.33%	71.01%
Lys	Glu	0.2904	0.2694	93.67%	90.64%
Thr	Asp	0.2177	0.1809	69.81%	65.23%
Tyr	Asp	0.0862	0.1461	48.48%	63.70%
Tyr	Glu	0.0952	0.1428	52.11%	63.66%
Asn	Asp	0.2069	0.1725	75.25%	66.34%
Gln	Asp	0.2380	0.2681	84.09%	83.06%
Ser	Glu	0.1415	0.1460	55.07%	59.44%

Donor	Acceptor	RSA_{donor}	$RSA_{acceptor}$	$P_{s,donor}$	$P_{s,acceptor}$
His	Asp	0.1417	0.1335	67.94%	58.29%
Arg	Asn	0.1890	0.1783	80.23%	63.69%
Asn	Asn	0.1680	0.2207	64.09%	71.43%
Arg	Gln	0.1901	0.1681	71.04%	65.21%
Asn	Glu	0.1478	0.1937	63.09%	71.24%
Gln	Glu	0.1952	0.2031	71.46%	77.85%
Arg	Ser	0.1634	0.1269	63.26%	54.88%
Thr	Glu	0.1535	0.1566	59.43%	56.13%
Arg	Thr	0.1716	0.1102	70.53%	48.07%
His	Glu	0.1460	0.1680	65.45%	65.45%
Arg	Tyr	0.1388	0.0681	64.10%	35.33%
Gln	Asn	0.2116	0.1990	75.29%	71.76%
Asn	Thr	0.1238	0.1539	55.52%	61.57%
Asn	Ser	0.1333	0.1680	61.82%	56.00%
Asn	Gln	0.1624	0.1531	65.93%	67.77%
Thr	Asn	0.1711	0.1911	59.70%	71.86%
Trp	Asp	0.0849	0.1379	44.12%	59.24%
Gln	Ser	0.1787	0.1439	64.73%	49.55%
Thr	Thr	0.1893	0.1822	69.06%	63.23%
Lys	Asn	0.2595	0.2434	79.72%	78.80%
Gln	Gln	0.1782	0.2286	71.23%	86.79%
Gln	Thr	0.1416	0.1468	54.03%	65.88%
Trp	Glu	0.0774	0.1294	49.28%	55.50%
Ser	Asn	0.1780	0.1937	53.14%	71.98%
Thr	Ser	0.1615	0.1414	55.15%	60.31%
Lys	Ser	0.1277	0.1165	43.45%	58.33%
Lys	Gln	0.3111	0.2466	95.21%	94.61%
Ser	Ser	0.1382	0.1399	53.80%	54.43%
Lys	Thr	0.2170	0.2542	93.20%	89.80%
Ser	Thr	0.1194	0.1144	48.92%	53.24%
Asn	Tyr	0.1362	0.0820	60.31%	46.56%
Gln	Tyr	0.1510	0.0777	63.93%	52.46%
Ser	His	0.1516	0.1385	61.54%	66.67%
Tyr	Asn	0.0582	0.1162	36.04%	67.57%
Thr	His	0.1994	0.1833	66.67%	68.52%
Tyr	His	0.0405	0.0567	18.87%	44.34%
Trp	Thr	0.0313	0.0598	24.04%	32.69%
Trp	Gln	0.0709	0.1164	35.05%	56.70%
Tyr	Gln	0.0664	0.1183	38.30%	50.00%
Thr	Gln	0.1417	0.1211	51.61%	55.91%
His	Thr	0.1185	0.1315	53.85%	45.05%
Ser	Gln	0.1415	0.1523	57.95%	60.23%
Tyr	Thr	0.0286	0.0607	12.64%	32.18%
His	Tyr	0.0682	0.0597	34.57%	28.40%
Trp	Tyr	0.0468	0.0445	32.10%	25.93%
Lys	His	0.2940	0.1557	83.54%	82.28%
Arg	His	0.1062	0.0802	41.67%	47.22%
His	Ser	0.0737	0.0960	54.93%	49.30%
Trp	Ser	0.0474	0.0563	22.54%	28.17%
Tyr	Ser	0.0362	0.0492	22.54%	19.72%
Lys	Tyr	0.2288	0.1079	72.86%	60.00%
His	Asn	0.1092	0.1391	62.50%	56.25%
Tyr	Tyr	0.0831	0.0683	45.16%	38.71%
Thr	Tyr	0.0610	0.0535	37.70%	31.15%
His	His	0.1159	0.1138	52.54%	50.85%
Trp	Asn	0.0889	0.1407	42.59%	62.96%
His	Gln	0.1763	0.1582	65.38%	75.00%
Asn	His	0.0400	0.0518	29.55%	34.09%
Gln	His	0.1286	0.1550	45.16%	87.10%
Trp	His	0.0355	0.0500	13.79%	27.59%

Donor	Acceptor	RSA_{donor}	$RSA_{acceptor}$	$P_{s,donor}$	$P_{s,acceptor}$
Ser	Trp	0.0000	0.0009	0.00%	0.00%
Ser	Tyr	0.0967	0.0637	39.29%	39.29%
Arg	Arg	0.2548	0.2092	92.31%	100.00%
Gln	Arg	0.2213	0.2265	66.67%	83.33%
Lys	Arg	0.2829	0.3459	100.00%	83.33%
Lys	Trp	0.2119	0.1800	100.00%	100.00%
Asn	Arg	0.2482	0.3328	66.67%	100.00%
Ser	Arg	0.1335	0.1290	50.00%	50.00%
Thr	Trp	0.0000	0.0412	0.00%	0.00%
Asn	Lys	0.5205	0.2538	100.00%	100.00%
His	Lys	0.1661	0.1237	100.00%	100.00%
Lys	Lys	0.3453	0.3839	100.00%	100.00%
Arg	Trp	0.2339	0.0004	100.00%	0.00%
Asn	Trp	0.0051	0.0526	0.00%	100.00%
Gln	Trp	0.0004	0.0137	0.00%	0.00%
Tyr	Trp	0.1072	0.4575	100.00%	100.00%

Table S7. Decomposition of interaction energies of the Tyr-Asp, Ser-Asp and Arg-Asp side chain-side chain hydrogen bonds at R = 2.55 Å. The energies are in the unit of kcal/mol. The total interaction energy of a hydrogen bond is partitioned into the frozen interaction, polarization and charge transfer energies. The frozen interaction energy is further decomposed into the contributions from the permanent electrostatics, Pauli repulsion and dispersion interactions in vacuum and a solvation energy (first four rows of the table). This decomposition is represented as $\Delta E^{(s)}_{TOT} = (\Delta E^{(v)}_{ELEC} + \Delta E^{(v)}_{PAULI} + \Delta E^{(v)}_{DISP} + \Delta E_{SOL}) + \Delta E^{(s)}_{POL} + \Delta E^{(s)}_{CT}$.

	Tyr-Asp	Ser-Asp	Arg-Asp
Electrostatics	-63.6	-53.4	-187.2
Pauli repulsion	64.3	55.6	121.1
Dispersion	-6.3	-5.0	-10.3
Solvation	10.8	8.7	101.2
Polarization	-7.0	-5.3	-14.5
Charge transfer	-11.1	-9.1	-22.4
Total interaction energy	-12.9	-8.5	-12.1

Table S8. The PBD IDs of the peptides and proteins in the training dataset. Each structure has a unique protein name.

1V0L	1C7K	1V6P	1VB0	1VBW	1VYR	1W23	1W3L	1W66	1W7J	1CC8	1WCG
1WY3	1X1K	1X6Z	1X8P	1XG0	1XQO	1Y55	1Y93	1YFQ	1YQS	1YS1	1Z2U
1ZK4	1ZL0	1ZLB	1ZLM	1ZUU	2A6Z	2A70	2AB0	2ABS	2AGY	2AIB	2AOG
2ASC	2AU7	2AVO	2AXW	2B3H	1CXQ	2B97	2BF6	2BF9	2BHU	2BOG	2BOI
2BT9	2BV4	2BZZ	2C2U	2C71	2CAL	2CAR	2CE2	2CHH	2CI1	2CNQ	2CWS
2CZQ	2D5M	2DDX	2DKO	2DSX	1D4T	2E3W	1D5T	2F01	2FBA	2FE5	2FMA
2FVY	2FWH	2G58	2G6F	2GG2	2GJ3	2GKG	2GUD	2H3L	2H5C	2H5O	2HEU
2HIN	2HS1	2HXS	2I5V	2II2	2IIM	2IXT	2J8T	1DS1	2JFR	2JHF	2NQH
2NRL	2NWF	2NXV	2NYB	2O7A	2O90	2OB3	2OIZ	1DY5	2OSX	2OV0	2P5K
2P74	2PL7	2PND	2PNE	2PPN	2PVB	2PWA	2QCP	2QOL	2QPN	2QSK	2QXI
2R0X	2R16	2R31	2RBK	2RH2	2RHF	2RK3	1EA7	2UU8	1EB6	2V3I	2V89
2V8F	2V8T	2V9V	2VB1	2VFR	2VHA	2VHK	2VHR	2VXN	2VZC	2VZP	2W15
2W39	2W72	2WBQ	2WF7	2WFI	1EJG	2WUR	2WW6	2X1Q	2X46	2X4K	2X5Y
2X9G	2XFR	2XJP	2XMJ	2XOD	2XOM	2XTT	2XU3	2XW6	2XWV	2Y61	2Y78
2YEO	2YHG	2YKZ	2Z4U	2Z72	2ZD8	1ET1	2ZPM	2ZQ7	1EUW	3A2O	3A4R
3A72	3AGN	3AJ4	1EXR	3AKQ	3AL1	3AUB	3AYJ	3AZD	3B64	3BF7	3BVA
3BVX	3BWH	3C6X	3C78	3CCD	3CIJ	3CT6	3CU9	3CUZ	3D1P	1F86	3D43
3DHA	3DJJ	3DK9	3E2O	3E4G	3E5T	3E7R	3E8M	3E8Y	3EA6	3EO6	3EWW
3EY6	3F1L	3F7L	3FIL	3FMU	3FSA	1AB1	3G21	3G46	3G5S	3G9X	3GMX
3GOE	3H31	3HGP	1FSG	3HNY	3I2Z	3I94	3IP0	3IQU	3JU4	3JUD	3JYO
3K34	3KFF	3KLR	3KWD	1G2Y	3L5L	3L8W	3LB2	1G4I	3LO8	3LQB	3LWX

1G66	3M5Q	3MCW	3MRE	1G8T	3MVS	1GA6	3NBC	3NE0	3NED	3NGP	3NON
3NOQ	1GCI	3NVS	3NYC	3O1C	3O4P	3O5Q	1AHO	3P4H	3P6D	3PB6	3PFZ
3PSM	3PUC	1GKM	3Q46	1GMX	3QL9	3QPA	3QZB	3QZR	3R2Q	3R40	1GQV
3RO3	3ROF	3RPE	3RQ9	3RWN	3RZN	3S6E	3SIL	3SK2	3SNF	3SOJ	3SU6
3SZH	1GWE	3T7L	3TC8	3TEU	3TG2	1GYN	3TRV	3TU8	3U7Q	3U8I	3UC7
3UE7	3UI4	3ULJ	3V0D	3V1E	3VHG	3VII	3VLA	3VN3	3VOR	3W07	3W42
3W5H	3W7Y	3WA2	3WAR	3WDN	3WGX	3WH1	3WOU	3X0I	3X1X	3X2M	3X34
3ZOJ	3ZQX	3ZR8	3ZSJ	3ZUC	3ZY7	3ZZP	4A02	4A29	4A7U	4A9V	4ACJ
4AFF	4ANN	4AOH	4AQO	4ATE	4AVR	4AWT	4AXO	4AYO	4B1M	4B5N	4B5O
4B9G	4BCT	1HJ8	1HJ9	4BLK	4BM1	4BPS	4BS0	4BT2	4BVM	4C1H	4CD5
4CE8	4CJ0	4CNG	4CNN	4CO3	4CVR	4CZ5	4D8B	4DP9	4E0K	4E3Y	4E9S
4EA9	4EGU	4EIC	4EIR	4EKF	4ES1	4ESP	4ETN	1I1W	4EUZ	1I27	4F1V
4FK9	4FKA	4G78	4G9E	4G9S	4GA2	4GJZ	4GNR	4GNU	4GS3	1UCS	4H4N
4H7W	4HE6	4HGU	4HKL	4HL2	4HNO	4HS1	4HUA	4HVU	4I62	4I8H	4IAU
1IGD	4ID0	4ID4	4IVV	4J1P	4J8C	4JED	4JER	4JOK	4JP6	4JZ5	4K12
4K6B	4K8M	4K8Y	4KEF	4KGD	4KQP	1ITX	4KXV	4L57	4LF0	4LFS	4LMM
4LN2	1IX9	1PEN	1IXH	4M51	4M7G	4M91	4M9V	4MAK	4MHP	4MIJ	1J0P
4MNC	4MPA	4MQ3	4MTU	4MX6	4MZC	4MZD	4N0K	4N1I	4N6X	4N7F	4NDS
4NSV	4O6U	4O8H	4OO4	4OP5	4OUS	4PE0	4PF3	4PLZ	4PSS	1JFB	4PZ3
4Q2L	4Q4G	4Q9W	4QA8	4QB3	4QI8	4QL3	4QLP	4QQS	4QRN	4QYT	4R2X
4R4S	4R5R	4RBX	4REK	4RJ2	4RQR	4RV5	4RXV	4TKB	4TXR	4TYF	4U2W
4U9H	4UA6	4UDX	4UE8	4UHC	4UNU	4URF	4UTJ	4UTM	4UYR	4UZG	4W7K
4W7W	4WEE	4WKA	4WPG	4WPK	4WUI	4WWF	4X2R	4X5P	4X6H	4X9Y	4XDX
4XOT	4XPX	4XUW	1K4I	4Y9W	4YAA	1K5C	1K5N	4YL4	4YMY	4YNH	4YPO
4YSI	4YTK	4ZA8	4ZA9	4ZGF	4ZO2	5A0Y	5A1I	5A71	5A8C	5A92	5ACM
5ADU	5AE0	5AGF	5AKR	1KF3	5AN7	5AOT	5AOU	5AQ0	5AQE	5AVD	5B8D
5BR4	5BT3	1KJQ	5CKL	5D5Y	5D7W	5D8V	5D9E	5DGJ	5DP2	1B0Y	5DZE
5E1Y	1KQP	5E7B	5E9N	5EKW	5ELB	5EM0	5EMB	1KT6	1KTH	5EWO	5F82
5F8S	5FAF	5FBF	5FG6	1KWF	5GJI	5GZC	5H5Q	5HB7	5HBS	5I40	5I86
5IDB	5IG6	5IHS	5IMA	5IQN	5IQX	5IVN	5IWH	5J1N	5J93	5JBX	1L3K
5JDT	5JH8	5JIG	5JK4	5JQF	5JUG	5LSV	5LVO	5M17	5M1P	1L9L	5PTI
5SV5	5SY4	5SY9	5TNV	5U4H	5WS7	7A3H	8A3H	1LNI	1LQT	1LS1	1LU0
1LUG	1LWB	1M1Q	1M2D	1M40	1M9Z	1MC2	1MFM	1MJ5	1MN8	1MSO	1MUW
1MWQ	1N62	1N9B	1NH0	1NKI	1NLS	1NNF	1NQJ	1NWZ	1O7J	1OAI	1OD3
1OE1	1OEW	1OH0	1OK0	1P1X	1P5F	1BKR	1PJX	1PM1	1PQ7	1PSR	1Q6Z
1BRF	1BS9	1QJ4	1QNJ	1QOW	1QTW	1QV0	1QXY	1R0R	1R2Q	1R6J	1RG8
1RTQ	1RW1	1RWY	1BXO	1S0Q	1BYI	1SBY	1BYZ	1SFS	1T8K	1TG0	1TJX
1TQG	1TT8	1U2H	1C5E	1UFY	1UG6	1UNQ	1UOW	1UOZ	1US0	1UWC	1C75
1UZ3	1UZV	2HBW	2V1M	3I06	1GVK	4CO8	4NI6	5FI3	1N40	1VRZ	1W0N
1W3M	1W7Q	1XMK	1XY1	1ZZK	1A0M	2ERL	2F4K	2IZQ	2NLS	2O6N	2O9S
2OL9	2V9B	2WQ1	2XJH	2Y5M	1A7Z	1ETL	3A02	3A0M	1F94	1G6X	3NIR
3NJW	3ODV	3Q8J	3QR7	3SBN	3TQ2	3U97	1AKG	3V1A	1ALZ	1HJE	4BPF
4D5M	1I2T	4HP2	4J9C	1IQZ	1ITT	4M6E	4NAG	4NPD	4OLR	1JBE	4PNO
4QK7	4RWC	4UBY	4UBZ	4W5L	4Z0W	5AL6	5I6A	5SVY	1LKK	1M24	1NKD
1OB6	1P9G										

Table S9. The PBD IDs of the peptides and proteins in the test dataset.

4Y9V	5B4J	5DJ7	5DK1	5DKM	5FLK	5GGB	5GV7	5GV8	5HGX	5HR3	5HUB
5HYV	5I4I	5I5B	5II6	5II8	5ILU	5J6Y	5JDK	5JSK	5JSY	5K1L	5K2Q
5KMW	5KWM	5KXV	5L2V	5L87	5LJP	5LJQ	5LJT	5LKV	5LL8	5LLC	5LP9
5LSH	5LUN	5LWS	5LWT	5M28	5M2T	5M4V	5M78	5MB3	5MB5	5MDU	5MH1
5MK9	5MN1	5MNB	5MNC	5MNE	5MNF	5MNG	5MNH	5MNK	5MNL	5MNM	5MNN
5MNO	5MNP	5MSZ	5MTU	5MYY	5N8B	5N8E	5N8J	5N8W	5N9H	5NC0	5NCG
5NE5	5NGX	5NKF	5NKG	5NLD	5NLH	5NQ0	5NVG	5NW3	5NWP	5NXV	5NXW
5NY1	5NY6	5NYK	5O2X	5O45	5O99	5OAV	5OAZ	5OBK	5OD4	5OF5	5OF6
5OFS	5OGJ	5OGN	5OGO	5OGP	5OHQ	5OJ5	5OK2	5OLR	5OME	5ONK	5OPF
5OTN	5OU0	5OUJ	5OUK	5OXC	5P9J	5P9O	5P9V	5QHT	5QHU	5QHV	5QI0
5QI1	5QI2	5QI3	5QI5	5QI6	5QI7	5QI8	5QI9	5SW1	5T39	5TDA	5TDB

5TIF	5TOG	5TVY	5U3A	5UMP	5VG0	5VLE	5W0G	5WAA	5WGI	5WQQ	5WQR
5X9L	5X9M	5XBU	5XBX	5XEC	5XP6	5XPF	5XPS	5XQP	5XQU	5XQV	5XR0
5XS6	5XSA	5XSB	5XSM	5XT4	5XTL	5XTV	5XTX	5XU2	5XUF	5XVT	5XYQ
5XYS	5Y0M	5Y2R	5Y2S	5Y9A	5Y9Z	5YCE	5YDE	5YOK	5YYP	5YYQ	5YYR
5Z3E	5Z3F	5ZGE	5ZGI	5ZGL	5ZGW	5ZGX	5ZGY	5ZGZ	5ZH0	5ZH1	5ZIO
5ZJ1	5ZJ2	5ZJ7	5ZJ8	5ZJC	5ZN1	5ZRC	5ZTD	5ZX8	6ADF	6AGN	6AIQ
6AIR	6AR0	6B00	6B8F	6BEV	6BG3	6BG5	6BQA	6BT6	6BWZ	6BXD	6C17
6C1J	6C1X	6C79	6C7A	6CDX	6CNU	6CNW	6CW7	6D1I	6DBC	6DCM	6DDJ
6DI1	6DQH	6DYF	6E1Z	6EIO	6EKZ	6ELV	6ENI	6ENP	6EQE	6ESM	6ETK
6ETL	6ETM	6ETN	6ETO	6ETP	6ETQ	6EUW	6EV6	6EV7	6EV8	6EVG	6EXX
6F1O	6F1R	6F54	6F7R	6F81	6F82	6F84	6F9O	6FGG	6FIH	6FIY	6FJ6
6FJ7	6FJN	6FJP	6FK2	6FM6	6FM7	6FMC	6FNG	6FNK	6FO5	6FPO	6FTF
6FVI	6FWG	6FWJ	6FWP	6G0V	6G1G	6G1I	6G3Q	6G7N	6GDC	6GGP	6GH7
6GKE	6GM9	6GX2	6GY5	6GZ8	6H10	6H40	6H5G	6HA3	6HAD	6HAV	6HMB
6HMC	6HMD	6HMH	6HMQ	6HMT	6HQX	6HR0	6HR3	6HRI	6HS9	6HSA	6HYO
6I03	6I0W	6I1U	6I3B	6I3E	6I74	6I7Y	6IC1	6IFP	6IGG	6IGH	6IIP
6IJY	6IXD	6J5S	6J64	6J93	6JGH	6JGI	6JGJ	6JGU	6JJ1	6JJQ	6JK4
6JKX	6JPT	6KBY	6KFN	6KGI	6KKZ	6KL0	6KL1	6KP5	6M7K	6M8F	6M8W
6M8Y	6MAP	6MM2	6MU0	6MU9	6MYE	6MZ1	6MZ2	6MZ3	6N3X	6N59	6NFR
6NI4	6NIA	6NJ3	6NNR	6O91	6OA7	6OWM	6P0Z	6P2L	6PWS	6Q2Y	6Q3P
6Q41	6Q48	6Q49	6Q4D	6Q4E	6Q4G	6Q4H	6Q4J	6Q4K	6Q6T	6Q7B	6Q7C
6Q7D	6Q7E	6Q7G	6QAZ	6QJK	6QJL	6QLN	6QLP	6QLR	6QLS	6QLU	6QRR
6QU6	6QW8	6QW9	6QWA	6QWB	6R1D	6R2Z	6R33	6R34	6R3N	6R4Z	6REK
6RGH	6RGP	6RHF	6RHH	6RHI	6RHO	6RHR	6RHU	6RHX	6RI0	6RI2	6RI4
6RI6	6RI8	6RII	6RJC	6RK0	6RO3	6RRV	6RT3	6RTP	6RUG	6RUH	6RUN
6RVG	6RYG	6RYN	6S07	6S14	6S17	6S1H	6S25	6S2M	6S2S	6S5W	6SID
6SP6	6TD0	6U66	6UKF	6UO3	6UQU	5HQI	5LHW	5NFK	5NFM	5NMN	5O0U
5OQZ	5VNY	5VSG	5WHN	5Y45	5Y46	6BTK	6C3T	6CHE	6DIX	6DKZ	6DL1
6E6O	6HG7	6I39	6M80	6N9H	6ODG	6Q5O	6Q5Q	6QLQ	6SYJ		

3. Supplementary figures

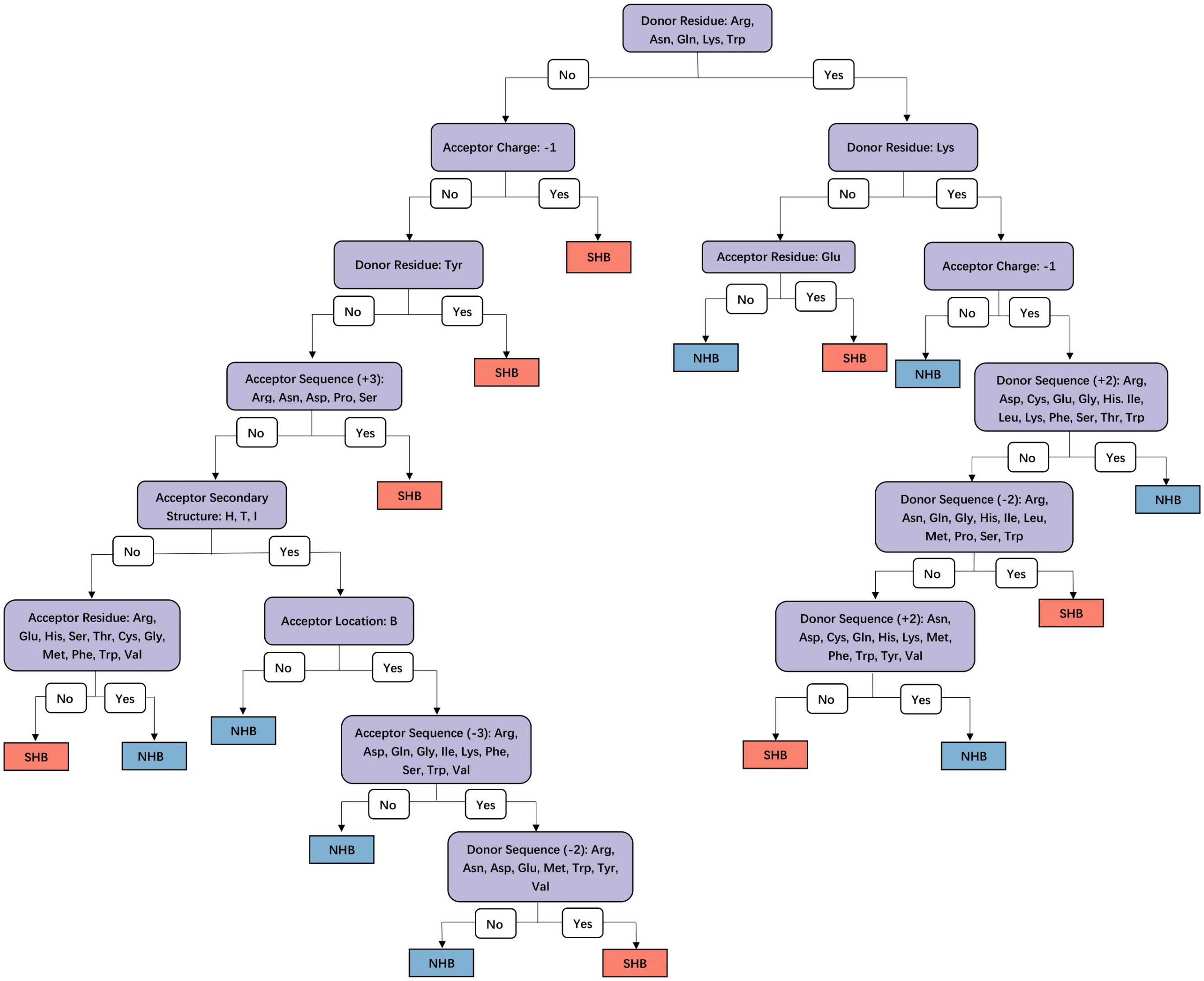

Fig. S1. An example decision tree model in the development of the MAPSHB model. The purple boxes represent the conditions of the judgements. The secondary structures that the hydrogen bond donor and acceptor residues can reside in include alpha helix (H), turn (T) and pi helix (I). From each balanced dataset, a boosting model is obtained from a weighted average of M such decision tree models (Algorithm 1). These decision trees are trained subsequently and each new model will put more weights on the data points that are predicted incorrectly in the previous step.

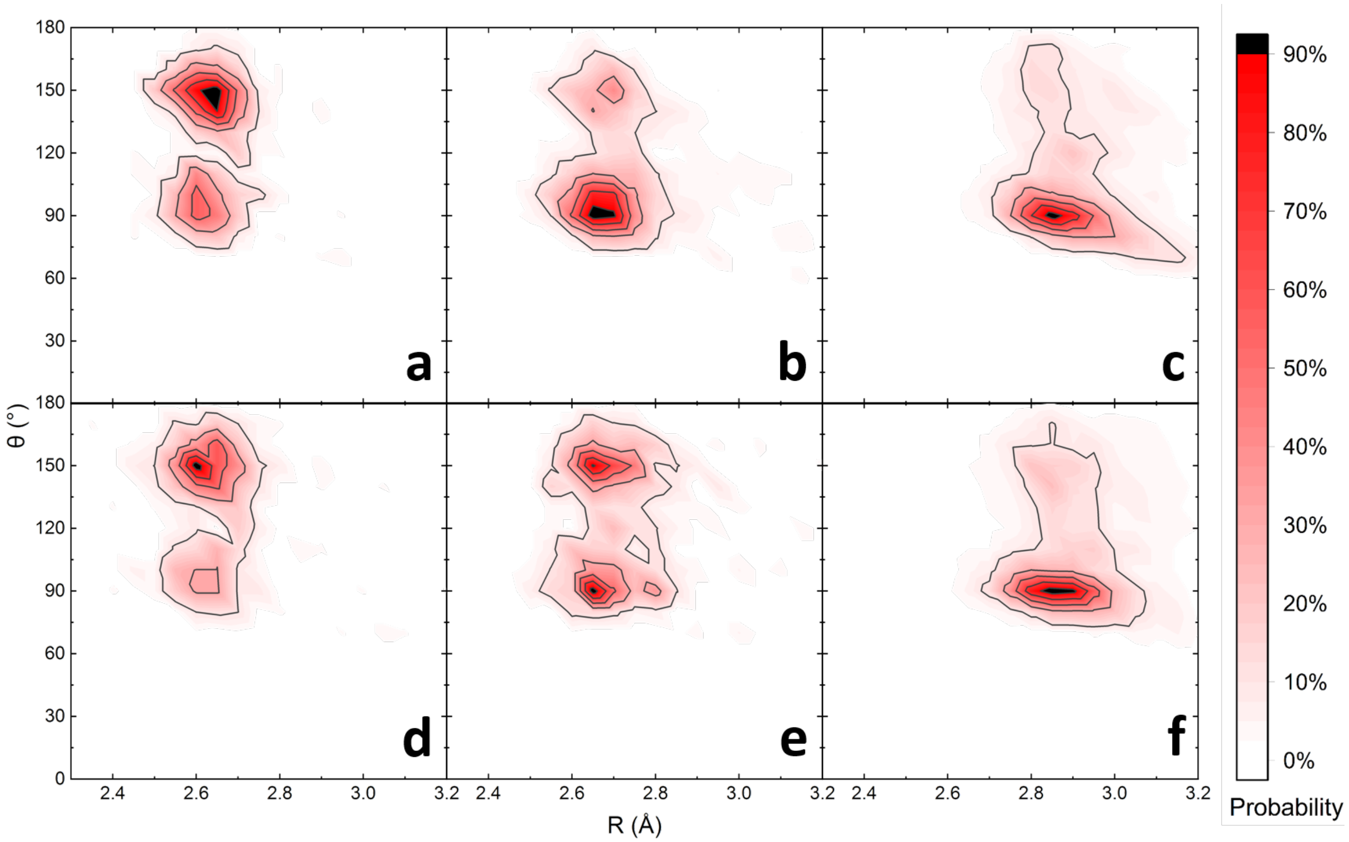

Fig. S2. The normalized distribution of $P(R, \theta)$ for the (a) Tyr-Asp, (b) Ser-Asp, (c) Arg-Asp, (d) Tyr-Glu, (e) Ser-Glu and (f) Arg-Glu side chain-side chain hydrogen bonds. The bin size is 0.05 Å for R and 10° for θ. The first maxima of the Tyr-Asp, Ser-Asp and Arg-Asp hydrogen bonds occur at (2.65 Å, 150°), (2.65 Å, 90°) and (2.85 Å, 90°), respectively. Their second maxima are at (2.60 Å, 100°), (2.70 Å, 150°) and (2.85 Å, 150°), respectively. The first maxima of the Tyr-Glu, Ser-Glu and Arg-Glu hydrogen bonds occur at (2.60 Å, 150°), (2.65 Å, 90°) and (2.85 Å, 90°), respectively. Their second maxima are at (2.60 Å, 90°), (2.65 Å, 150°) and (2.85 Å, 140°), respectively. In a-f, the ratio between the second and first maxima are 56%, 42%, 14%, 32%, 93% and 23%, respectively.

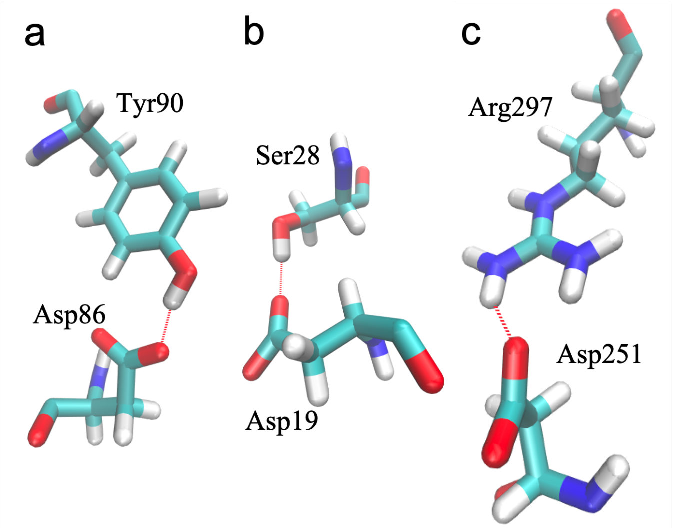

Fig. S3. Representative configurations of the Tyr-Asp, Ser-Asp and Arg-Asp side chain-side chain hydrogen bonds as obtained from the second maxima of their geometric probability distributions. (a) A SHB between Tyr90 and Asp86 (R = 2.60 Å) in a β-carboxysomal γ-carbonic anhydrase varient (PDB ID 3KWD).[19] (b) A SHB between Ser28 and Asp19 (R = 2.70 Å) in a green fluorescent protein mutant (PDB ID 2WUR).[20] (c) A NHB between Arg297 and Asp251 (R = 2.85 Å) in a histidine ammonia-lyase (PDB ID 1GKM).[21]

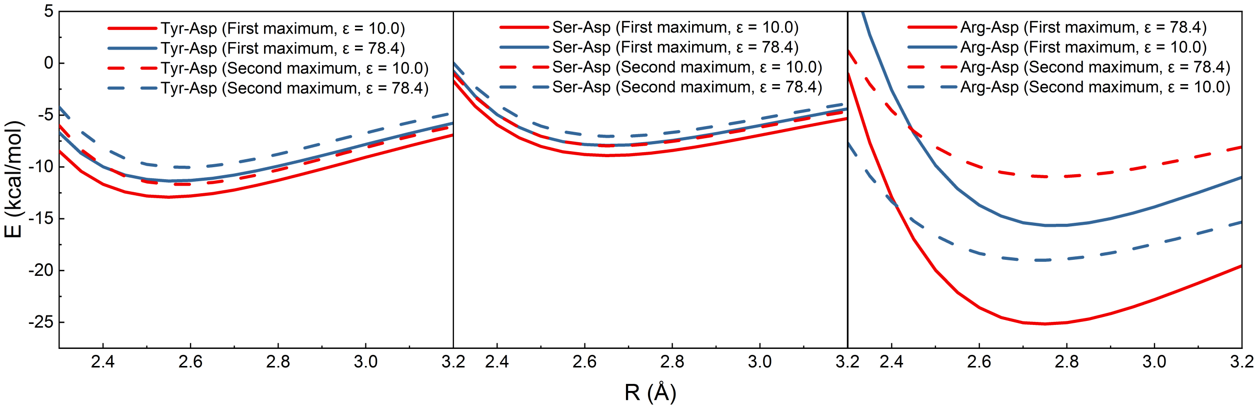

Fig. S4. Hydrogen bond energies for different configurations and solvation environments of the Tyr-Asp, Ser-Asp and Arg-Asp side chain-side chain hydrogen bonds when R is between 2.3 Å and 3.2 Å. For each pair, the red and blue solid lines are the potential energy surfaces for the configuration from the first maximum of their geometric probability distributions (Fig. 3) in different solvation conditions. Similarly, the red and blue dashed lines are the potential energy surfaces for the configuration from the second maximum of the probability distributions (Fig. S3) in different solvation conditions. The potential energy surfaces of the hydrogen bond interactions are computed from electronic structure calculations by keeping the relative orientation of the donor and acceptor groups and changing R of the hydrogen bond. Here we use a dielectric constant of $\varepsilon = 10.0$ in the PCM model to represent a protein environment around a hydrogen bond, and $\varepsilon = 78.4$ to represent an aqueous solution environment. Comparing the red and blue (solid or dashed) curves, the differences in interaction energies are 1.1-1.8 kcal/mol for the Tyr-Asp hydrogen bond, 0.8-1.0 kcal/mol for the Ser-Asp hydrogen bond, and 7.2-10.5 kcal/mol for the Arg-Asp hydrogen bond at all R values. For each hydrogen bond pair, the most probable configuration has a lower energy than the second most probable structure. Among the three cases, the Arg-Asp pair is most sensitive to the solvation environment and we choose an ε of 78.4 since it has a high probability of solvent exposure.

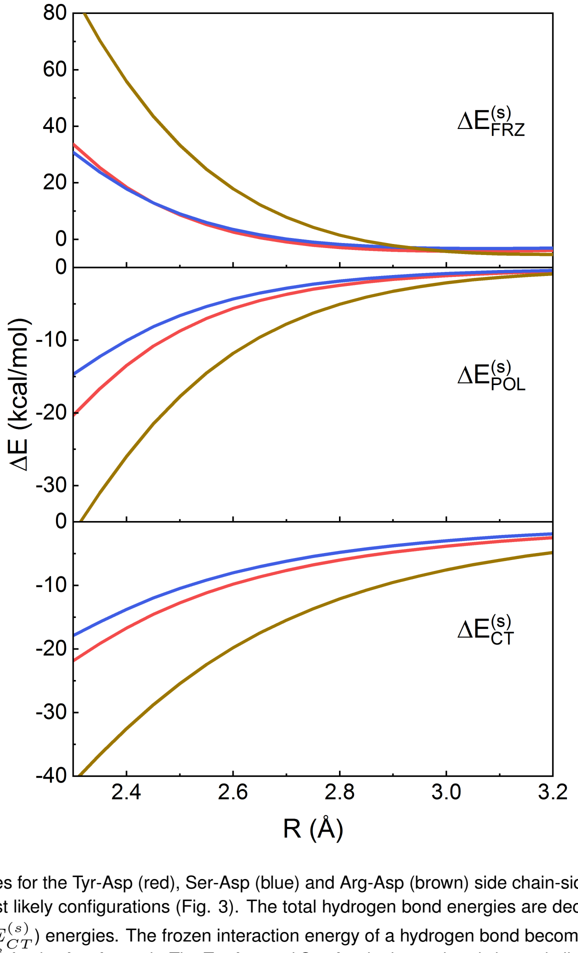

Fig. S5. Decomposition of the interaction energies for the Tyr-Asp (red), Ser-Asp (blue) and Arg-Asp (brown) side chain-side chain hydrogen bonds when R is between 2.3 Å and 3.2 Å. The hydrogen bonds take their most likely configurations (Fig. 3). The total hydrogen bond energies are decomposed into the frozen interaction ($\Delta E^{(s)}_{FRZ}$), polarization ($\Delta E^{(s)}_{POL}$) and charge transfer ($\Delta E^{(s)}_{CT}$) energies. The frozen interaction energy of a hydrogen bond becomes negative when R is larger than 2.70 Å for the Tyr-Asp pair, 2.75 Å for the Ser-Asp pair and 2.85 Å for the Arg-Asp pair. The Tyr-Asp and Ser-Asp hydrogen bonds have similar frozen interaction energies, and their differences are around 1 kcal/mol when $R > 2.40$ Å. The differences in polarization and charge transfer energies can be larger than 3 kcal/mol between the two cases. The Arg-Asp hydrogen bond has larger energy components compared to the other two cases as both its donor and acceptor residues are charged.

References

1. Huang, Y., Niu, B., Gao, Y., Fu, L. & Li, W. CD-HIT Suite: a web server for clustering and comparing biological sequences. *Bioinformatics* **26**, 680–682 (2010).
2. Case, D. A. *et al.* The Amber biomolecular simulation programs. *J. Comput. Chem.* **26**, 1668–1688 (2005).
3. Kabsch, W. & Sander, C. Dictionary of protein secondary structure: Pattern recognition of hydrogen-bonded and geometrical features. *Biopolymers* **22**, 2577–2637 (1983).
4. He, H. & Garcia, E. A. Learning from imbalanced data. *IEEE Transactions on Knowl. Data Eng.* **21**, 1263–1284 (2009).
5. Bishop, C. M. *Pattern Recognition and Machine Learning (Information Science and Statistics)* (Springer-Verlag, Berlin, Heidelberg, 2006).
6. Mitternacht, S. FreeSASA: An open source C library for solvent accessible surface area calculations. *F1000Research* **5**, 189 (2016).
7. Lee, B. & Richards, F. The interpretation of protein structures: Estimation of static accessibility. *J. Mol. Biol.* **55**, 379 – 400 (1971).
8. Tien, M. Z., Meyer, A. G., Sydykova, D. K., Spielman, S. J. & Wilke, C. O. Maximum allowed solvent accessibilites of residues in proteins. *PLoS ONE* **8**, e80635 (2013).
9. Rose, G. D., Geselowitz, A. R., Lesser, G. J., Lee, R. H. & Zehfus, M. H. Hydrophobicity of amino acid residues in globular proteins. *Science* **229**, 834–838 (1985).
10. Miller, S., Janin, J., Lesk, A. M. & Chothia, C. Interior and surface of monomeric proteins. *J. Mol. Biol.* **196**, 641 – 656 (1987).
11. Porollo, A. & Meller, J. Prediction-based fingerprints of protein–protein interactions. *Proteins* **66**, 630–645 (2007).
12. Zellner, H. *et al.* Prescont: Predicting protein-protein interfaces utilizing four residue properties. *Proteins* **80**, 154–168 (2012).
13. Lucas, X., Bauzá, A., Frontera, A. & Quiñonero, D. A thorough anion–π interaction study in biomolecules: on the importance of cooperativity effects. *Chem. Sci.* **7**, 1038–1050 (2016).
14. Becke, A. D. Density-functional thermochemistry. III. The role of exact exchange. *J. Chem. Phys.* **98**, 5648 (1993).
15. Witte, J., Mardirossian, N., Neaton, J. B. & Head-Gordon, M. Assessing dft-d3 damping functions across widely used density functionals: Can we do better? *J. Chem. Theory Comput.* **13**, 2043–2052 (2017).
16. Warwicker, J. pKa predictions with a coupled finite difference Poisson–Boltzmann and Debye–Hückel method. *Proteins* **79**, 3374–3380 (2011).
17. Li, L., Li, C., Zhang, Z. & Alexov, E. On the dielectric "constant" of proteins: Smooth dielectric function for macromolecular modeling and its implementation in DelPhi. *J. Chem. Theory Comput.* **9**, 2126–2136 (2013).
18. Vennelakanti, V., Qi, H. W., Mehmood, R. & Kulik, H. J. When are two hydrogen bonds better than one? accurate first-principles models explain the balance of hydrogen bond donors and acceptors found in proteins. *Chem. Sci.* **12**, 1147–1162 (2021).
19. Peña, K. L., Castel, S. E., de Araujo, C., Espie, G. S. & Kimber, M. S. Structural basis of the oxidative activation of the carboxysomal γ-carbonic anhydrase, CcmM. *Proc. Natl. Acad. Sci. U.S.A.* **107**, 2455–2460 (2010).
20. Shinobu, A., Palm, G. J., Schierbeek, A. J. & Agmon, N. Visualizing proton antenna in a high-resolution green fluorescent protein structure. *J. Am. Chem. Soc.* **132**, 11093–11102 (2010).
21. Baedeker, M. & Schulz, G. E. Structures of two histidine ammonia-lyase modifications and implications for the catalytic mechanism. *Eur. J. Biochem.* **269**, 1790–1797 (2002).
